# Electronic and Optical Properties of 2D Heterostructure Bilayers of Graphene, Borophene and 2D Boron Carbides from First Principles

**DOI:** 10.3390/nano14201659

**Published:** 2024-10-16

**Authors:** Lu Niu, Oliver J. Conquest, Carla Verdi, Catherine Stampfl

**Affiliations:** 1 School of Physics, The University of Sydney, Sydney, NSW 2006, Australia; 2 School of Mathematics and Physics, The University of Queensland, Brisbane, QLD 4072, Australia

**Keywords:** graphene, borophene, BC3, B4C3, two-dimensional, heterostructure, van der Waals, DFT, optical, dielectric

## Abstract

In the present work the atomic, electronic and optical properties of two-dimensional graphene, borophene, and boron carbide heterojunction bilayer systems (Graphene–BC_3_, Graphene–Borophene and Graphene–B_4_C_3_) as well as their constituent monolayers are investigated on the basis of first-principles calculations using the HSE06 hybrid functional. Our calculations show that while borophene is metallic, both monolayer BC_3_ and B_4_C_3_ are indirect semiconductors, with band-gaps of 1.822 eV and 2.381 eV as obtained using HSE06. The Graphene–BC_3_ and Graphene–B_4_C_3_ bilayer heterojunction systems maintain the Dirac point-like character of graphene at the K-point with the opening of a very small gap (20–50 meV) and are essentially semi-metals, while Graphene–Borophene is metallic. All bilayer heterostructure systems possess absorbance in the visible region where the resonance frequency and resonance absorption peak intensity vary between structures. Remarkably, all heterojunctions support plasmons within the range 16.5–18.5 eV, while Graphene–B_4_C_3_ and Graphene–Borophene exhibit a π-type plasmon within the region 4–6 eV, with the latter possessing an additional plasmon at the lower energy of 1.5–3 eV. The dielectric tensor for Graphene–B_4_C_3_ exhibits complex off-diagonal elements due to the lower P3 space group symmetry indicating it has anisotropic dielectric properties and could exhibit optically active (chiral) effects. Our study shows that the two-dimensional heterostructures have desirable optical properties broadening the potential applications of the constituent monolayers.

## 1. Introduction

Graphene is well-known to have excellent electrical conductivity, desirable mechanical and thermal properties, and high light transmittance in the visible light–infrared region. It also has an extremely high quantum efficiency for light-matter interactions and is strongly optically nonlinear. It has therefore found applications in e.g., electronics, energy storage, biomedical and other fields [1]. Relative to conventional plasmonic materials, graphene possesses highly confined plasmons with much longer lifetimes. Also, graphene plasmons are active in an extended wavelength range, namely, the mid-infrared and terahertz regime. This wavelength range overlaps with the feature signals of most organic and biomolecules, which has broadened graphene’s applications towards plasmonic biological and chemical sensors [2] in addition to plasmonic waveguides, and infrared photodetectors. Moreover, the properties can be tuned by forming graphene-hybrid materials, finding applications in biosensors, chemical sensors, optical sensors, and other types of sensors [3].

In other areas, however, the use of graphene has been limited due to its zero-value band gap. One of the methods used to expand the application of graphene is to form heterostructures. Stacking different two-dimensional (2D) materials together can form double-layer or even multi-layer artificial materials that are maintained by van der Waals interactions [4]. Such materials are known as van der Waals heterojunctions. A wide range of physical properties can be obtained by such stacking, making van der Waals heterojunctions even more important than the 2D material itself. There are vast numbers of 2D materials, and with forming heterostructures thereof, a diverse range of electrical, photonic, plasmonic, mechanical, and chemical properties can be theoretically predicted through ab initio calculations and machine learning [5,6], and experimentally engineered [7,8].

Recently, the prospect of developing plasmonic devices employing 2D semiconductors has attracted considerable attention [9,10]. Plasmonic modes in each class of van der Waals semiconductors have their own peculiarities, along with potential technological capabilities. Recently, graphene-like BC_3_ has been synthesized experimentally [11,12,13] and is found to be semiconducting with an indirect band gap. Calculations have shown that nanostructures of graphene-like BC_3_ possess desirable absorbance in the visible region and by changing the size of the nanostructure, the resonance peak position of the absorption spectrum can be effectively regulated [14]. Recent studies have also predicted a stable B_4_C_3_ monolayer which is even more stable than the previously synthesized BC_3_ monolayer [15]. Boron carbides are known for their exceptional hardness, thermal stability, and chemical inertness. The emergence of 2D boron carbides adds a new dimension to the study of these materials, offering novel properties and opportunities for technological innovation [16,17].

Borophene is another 2D material of high current interest. This monolayer allotrope of boron forms several structures and is characterized by its unique atomic structure and exceptional electronic properties, which has opened new avenues for innovation in the areas of electronics, energy storage, catalysis, plasmonics, superconductivity, and sensors [11]. One particularly intriguing aspect of borophene’s versatility lies in its ability to form heterojunctions—interfaces between different borophene structures or between borophene and other materials. Borophene-graphene heterostructures have been realized [18] where they have shown good stability and ability to function as a humidity sensor, exhibiting a sensitivity about 700 times higher than that of pristine graphene, and 27 times higher than that of borophene. Also, recently both lateral and vertical integration of borophene with graphene has been achieved [19], as has the formation of bi-layer (α-phase) borophene on the Ag(111) surface [20].

The synergistic combination of graphene’s exceptional electrical conductivity, mechanical strength, and flexibility with boron carbide’s high hardness, thermal stability, and chemical resistance, and with borophene’s attractive electronic properties, hold tremendous potential for a wide range of applications spanning from electronics and optoelectronics to energy storage and sensing. As research in this field continues to advance, the development of scalable synthesis methods, the elucidation of structure-property relationships, and the exploration of novel applications will be key focal points. To date, there is little known about the physical properties of graphene-based bilayer heterojunctions with borophene and boron-carbide materials. This motivates the present work to gain an understanding of these heterojunctions, through the study of their electronic and optical properties on the basis of first-principles calculations. Such understanding is crucial for their future technological application, where the unique properties of these materials can be harnessed.

The paper is organised as follows: in Section 2 the calculation method is described, followed by the Results and Discussion in Section 3. Herein we describe the physical properties of the monolayers and heterostructures, including band structure, density of states, electron density difference distributions, Schottky barriers and the optical properties. This is followed by the Conclusions in Section 4.

## 2. Calculation Method

We perform first-principle calculations using density functional theory (DFT) as implemented in the Vienna Ab initio Simulation Package (VASP) [21,22,23]. The projector augmented wave (PAW) method pseudopotentials are used to account for the electron-ion interactions [24]. For the structural optimization calculations, we use the generalized gradient approximation (GGA) of Perdew-Burke-Ernzerhof (PBE) as the exchange-correlation functional [25]. The Becke-Johnson damped D3(BJ) dispersion correction is also included to account for long-range van der Waals (vdW) interactions [26,27,28]. A Γ-centered **k**-point sampling mesh of 11×11×1 (23×23×1 for the smaller graphene unit cell, see Figure S1) and a plane wave energy cutoff of 450eV are used. Electronic convergence criteria are set to $10^{-6}$ eV and systems are allowed to relax until all forces on each atom are below 0.01 eV/$\overset{˚}{A}$. For smearing of the electronic states we used the Gaussian smearing method implemented in VASP.

For the calculation of electronic properties, the PBE exchange-correlation functional is known to underestimate band-gap energies so we also use the screened hybrid exchange-correlation functional of Heyd, Scuseria, and Ernzerhof (HSE06) which gives more accurate band-gap energies [29,30,31]. All density of states (DOS) calculations use a Γ-centered **k**-point sampling mesh of 33×33×1 and Gaussian smearing of the electronic states with a broadening value of 0.1 eV. The HSE06 exchange-correlation functional is also used for the calculation of optical properties, in particular, the frequency-dependent dielectric function with a Γ-centered **k**-point sampling mesh of 17×17×1 and 65×65×1. The complex frequency-dependent dielectric function, ϵ(ω), is given by Equation (1), where $\epsilon_{1}\left(\omega \right)$ and $\epsilon_{2}\left(\omega \right)$ are the real and imaginary parts, respectively, and ω is the frequency in eV:

(1) $$\epsilon \left(\omega \right)=\epsilon_{1}\left(\omega \right)+i\epsilon_{2}\left(\omega \right)$$

In Equation (1) the imaginary part of the dielectric function, $\epsilon_{2}\left(\omega \right)$, is given by:

(2) ϵ2(ω)=Im ϵαβ(ω)=4π2e2Ωlimq→01q2∑c,v,k2ωkδ(Eck−Evk−ω)×〈μck+eαq|μvk〉〈μvk|μck+eβq〉

where k is the electron wavevector, $\omega_{k}$ is the k-point weight, *q* is the Bloch vector for the incident wave and Ω is the unit cell volume [32,33]. Here *v* and *c* are the valence and conduction bands indices, respectively. The $\mu_{vk/ck}$ terms are the cell periodic parts of the orbitals and $e_{\alpha /\beta }$ are the unit vectors for the three Cartesian directions. Lastly, $E_{ck}$ is the $c^{\mathrm{th}}$ conduction band energy and $E_{vk}$ is the $v^{\mathrm{th}}$ valence band energy.

The real part of the dielectric function is obtained by applying the Kramers-Kronig transformation to the imaginary part of the dielectric function in Equation (2):

(3) ϵ1(ω)=Reϵαβ(ω)=δαβ+2πP∫0∞dω′ω′ϵ2(ω′)ω′2−ω2+iη

where *P* is the principal value and η (here η=0.1) is the complex shift.

Using the dielectric function we also calculate the absorption coefficient, α(ω), given in Equation (4), where *c* is the speed of light.

(4) $$\alpha \left(\omega \right)=\frac{\sqrt{2}\omega }{c}{\left[{\left(\epsilon_{1}^{2}\left(\omega \right)+\epsilon_{2}^{2}\left(\omega \right)\right)}^{\frac{1}{2}}-\epsilon_{1}\left(\omega \right)\right]}^{\frac{1}{2}}$$

The loss function, L(ω), is calculated using the expression:

(5) $$L\left(\omega \right)=\frac{\epsilon_{2}\left(\omega \right)}{\epsilon_{1}^{2}\left(\omega \right)+\epsilon_{2}^{2}\left(\omega \right)}$$

For the calculation of the refractive index n(ω), extinction coefficient k(ω) and reflectivity R(ω) we use Equations (6), (7) and (8), respectively. In Equation (8), *n* is the refractive index and *k* is the extinction coefficient.

(6) $$n\left(\omega \right)={\left[\frac{\sqrt{\epsilon_{1}^{2}+\epsilon_{2}^{2}}+\epsilon_{1}}{2}\right]}^{\frac{1}{2}}$$

(7) $$k\left(\omega \right)={\left[\frac{\sqrt{\epsilon_{1}^{2}+\epsilon_{2}^{2}}-\epsilon_{1}}{2}\right]}^{\frac{1}{2}}$$

(8) $$R\left(\omega \right)=\frac{{\left(n-1\right)}^{2}+k^{2}}{{\left(n+1\right)}^{2}+k^{2}}$$

We note that the dielectric tensor contains the combined dielectric response of the 2D material and the vacuum so that the magnitude of $\epsilon_{1}$ and $\epsilon_{2}$ depend linearly on the thickness of the vacuum [34,35]. In the present work, for all systems, the vacuum region is 11 $\overset{˚}{A}$.

The interlayer binding energies ($E_{b}$) of the bilayer heterostructures are calculated using Equation (9), where $E_{H}$ is the total energy of the heterostructure, $E_{\mathrm{BC}/B}$ is the total energy of the isolated boron-carbide (/borophene) monolayer and $E_{G}$ is the total energy of the Graphene monolayer.

(9) $$E_{b}=E_{H}-E_{\mathrm{BC}/B}-E_{G}$$

The charge density difference (Δρ(r)) for the heterostructure systems is calculated using Equation (10). The $\rho_{\mathrm{AB}}\left(r\right)$ term is the total charge density of the heterostructure system and $\rho_{A/B}\left(r\right)$ are the total charge densities of the isolated monolayers making up the heterostructure.

(10) $$\Delta \rho \left(r\right)=\rho_{\mathrm{AB}}\left(r\right)-\rho_{A}\left(r\right)-\rho_{B}\left(r\right)$$

## 3. Results and Discussion

### 3.1. Monolayer Properties

**Figure 1:**
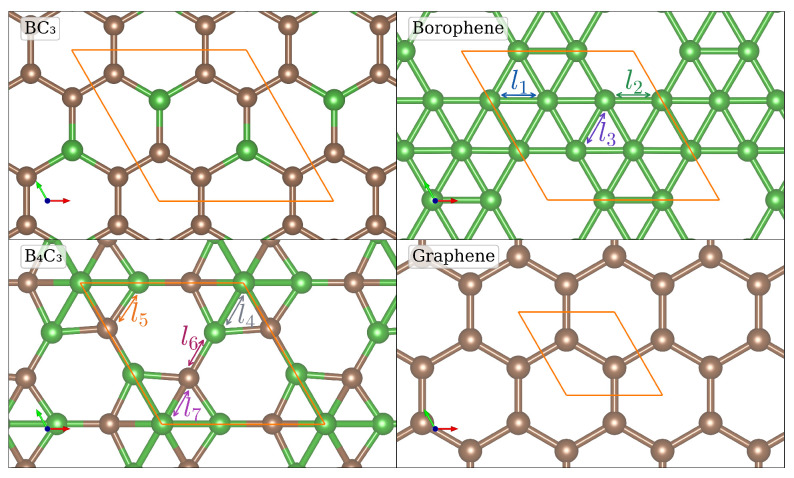
Optimized atomic structures of BC_3_, borophene, B_4_C_3_ and graphene. Boron and carbon atoms are denoted by the green and brown spheres, respectively. Borophene has three unique bonds indicated by $l_{1}$, $l_{2}$ and $l_{3}$, while B_4_C_3_ has four unique bonds indicated by $l_{4}$, $l_{5}$, $l_{6}$ and $l_{7}$. The unit cells are highlighted in orange.

We start by investigating the structural and electronic properties of the three monolayers, namely BC_3_, B_4_C_3_ and borophene. The graphene monolayer is also investigated as it always forms one layer of the heterostructure systems. The optimized structures of BC_3_, B_4_C_3_, borophene and graphene are shown in Figure 1. The monolayer allotrope of borophene has been predicted to have various low-energy structures with the $\beta_{12}$, $\chi_{3}$ and $\alpha^{\prime }$ structures having been synthesised experimentally [36]. For this study we chose to investigate the $\alpha^{\prime }$ structure of borophene, not only because it has been synthesised experimentally, but also because it has been shown to have the most favourable cohesive energy from GGA level ab initio calculations [37].

**Figure 2:**
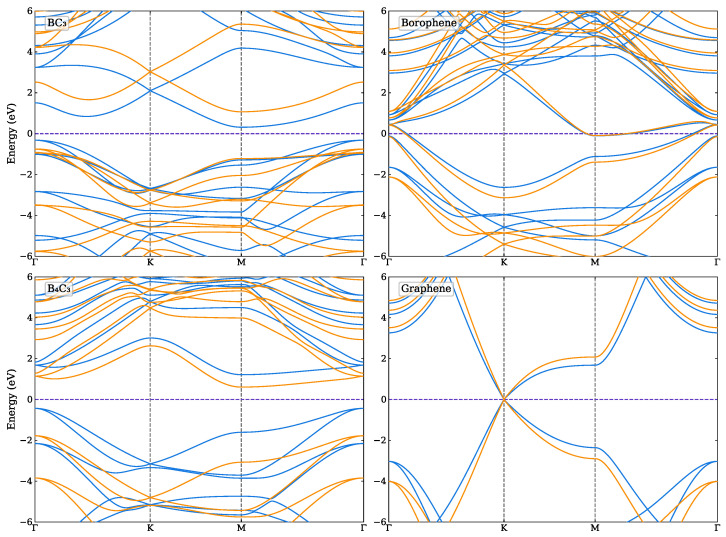
Band structure of the four monolayer systems, BC_3_, B_4_C_3_, borophene and graphene as calculated using the PBE (blue) and HSE06 (orange) functionals. The Fermi level is indicated by the purple dashed line.

The structural properties and band gap energies of the monolayer systems are shown in Table 1. For graphene the lattice constant is calculated to be a=2.467 $\overset{˚}{A}$, while those of borophene, BC_3_ and B_4_C_3_ are 5.051 $\overset{˚}{A}$, 5.169 $\overset{˚}{A}$ and 4.690 $\overset{˚}{A}$, respectively. In agreement with previous calculations, graphene is a semi-metal, borophene is metallic, while BC_3_ and B_4_C_3_ are indirect semiconductors [38,39] with calculated indirect band-gaps of 1.822 eV (0.634 eV) and 2.381 eV (1.647 eV) as obtained by the HSE06 (PBE), respectively. In Figure S2 the optimisation of the lattice constants for the monolayers are shown and the band structures of the four monolayer systems are shown in Figure 2.

### 3.2. Heterostructures

#### 3.2.1. Structural Properties

We now turn to determining the structure of the bilayer heterojunctions, which are composed of monolayer graphene stacked with monolayer BC_3_, borophene, and B_4_C_3_ (namely, Graphene–BC_3_, Graphene–Borophene and Graphene–B_4_C_3_), respectively. To create the heterostructues we use a (2×2) cell. For each system, we considered various lateral coordinations (positions) of these layers. Specifically, “Hollow”, “Bridge” and “Top” coordinations for the Graphene–BC_3_ bilayer systems, and “Hollow 1”, “Hollow 2”, “Bridge” and “Top” coordinations for the Graphene–Borohene and Graphene–B_4_C_3_ bilayer systems. We studied the relative energies, optimized lattice constants, and inter-planar distances for all structures. Details about this procedure are given in Figures S3–S6. Due to the weak van der Waals interaction between the layers, the difference in energies of the lateral positions is very small, with the values given in Table S1.

**Figure 3:**
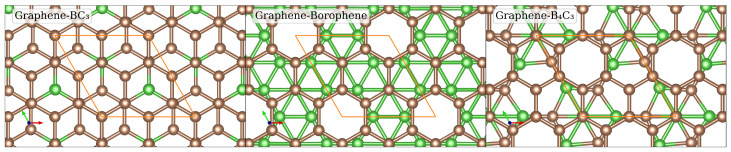
Top view of the optimized atomic structures of Graphene–BC_3_, Graphene–Borophene, and Graphene–B_4_C_3_. Boron and carbon atoms are denoted by the green and brown spheres, respectively. The unit cell is indicated by the orange parallelogram.

The results show that the energetically most favourable lateral position for the Graphene–BC_3_ system is the “Hollow” coordination stacking configuration. This means that each atom in BC_3_ is projected onto the center of a hexagon in graphene as shown in Figure 3. The bilayer system Graphene–Borophene and Graphene–B_4_C_3_ have the “Top” coordination stacking configuration as the energetically most favourable one. In this arrangement, each hexagon without an atom in the bottom layer is projected onto the center of a hexagon in graphene, but not every hexagon center in graphene aligns with a hexagon without an atom from the bottom layer (see Figure 3).

It can be seen from Table 2 that Graphene–Borophene is metallic, while Graphene–BC_3_ and Graphene–B_4_C_3_ are semi-metals. Out of all the heterostructure systems, a maximum (tensile) strain of 3.33% is present for B_4_C_3_. For the bilayer system Graphene–BC_3_, graphene is under tensile strain and BC_3_ is under compressive strain. For the bilayer system Graphene–Borophene, a slight tensile strain is present for Graphene, while borophene experiences compressive strain. For the bilayer system Graphene–B_4_C_3_, there is compressive strain in Graphene, while B_4_C_3_ is under a larger tensile strain. Generally, tensile strain tends to narrow the band gap, reducing it and increasing conductivity [43]. Conversely, compressive strain tends to widen the band gap, thereby decreasing conductivity [44].

#### 3.2.2. Charge Density Difference

**Figure 4:**
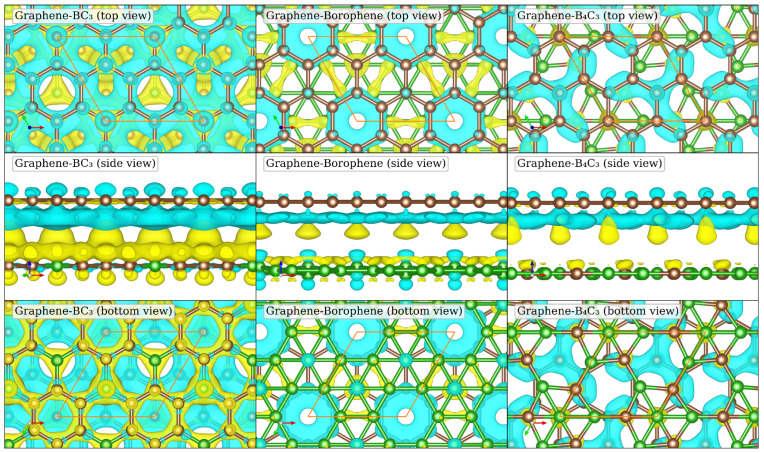
Charge density difference (Δρ(r), calculated using Equation (10)) between the monolayers and the heterostructures. Regions of charge accumulation are shown in yellow and regions of charge depletion are shown in blue. The isosurface level is $1.5\times 10^{-4}$ a_0_^−3^ and the top layer is always graphene. The unit cell is indicated by the orange parallelogram.

The charge density difference distributions for the heterostructure systems are shown in Figure 4 as calculated using Equation (10) by subtracting the charge density of the monolayers from the charge density of the heterostructure system. It is clear from the side view (middle panels) in Figure 4 that in all cases there is charge depletion from the graphene layer (top layer) and accumulation in the alternate monolayer. The largest charge redistribution is found for Graphene–BC_3_. This is consistent with the smaller interlayer distance, due to the hollow site relative coordination rather than the top site, as for the Graphene–Bororphene and Graphene–B_4_C_3_ heterostructures. We also observe that atoms in the lower layers which directly coordinate with a carbon atom in the graphene layer have the largest charge accumulation regions. In contrast, the carbon atoms in the graphene layer with bridge or hollow coordination to the atoms of the lower layer show the largest regions of charge depletion. This gives rise to the patterns in the charge accumulation/depletion regions shown in the top and bottom views of Figure 4. Therefore, we find the relative coordination between the bilayer heterostructures has a direct impact on their electronic properties. For the Graphene–B_4_C_3_ heterostructure, quite striking is the 3-fold rotational symmetry as seen from the top and bottom views (upper and lower plots of the rightmost panel).

In the next sections the band structure and density of states are investigated to further characterise the electronic structure of these heterostructure systems.

#### 3.2.3. Band Structure and Density of States

**Figure 5:**
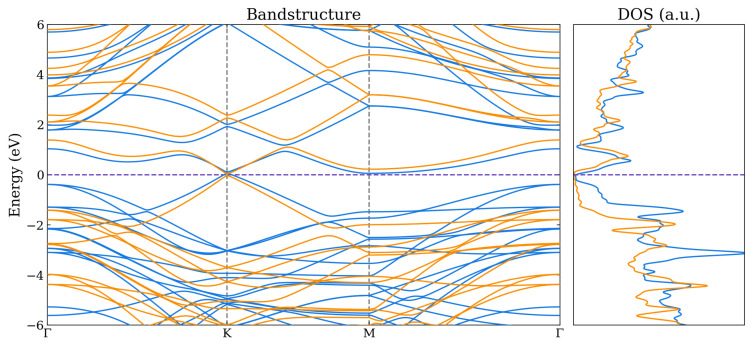
Band structure and total DOS for Graphene–BC_3_ as calculated using the PBE (blue) and HSE06 (orange) functionals. The Fermi level is indicated by the purple dashed line.

**Figure 6:**
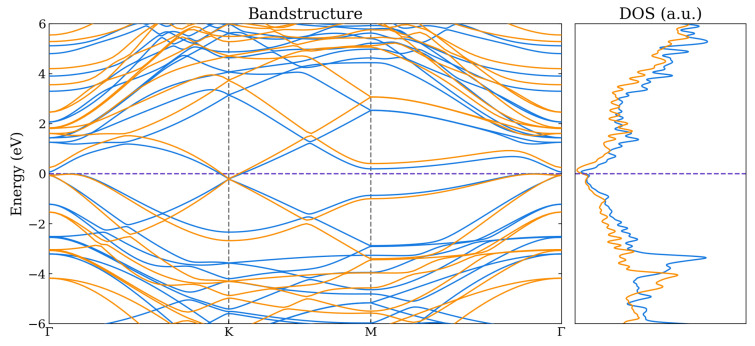
Band structure and total DOS for Graphene–Borophene as calculated using the PBE (blue) and HSE06 (orange) functionals. The Fermi level is indicated by the purple dashed line.

**Figure 7:**
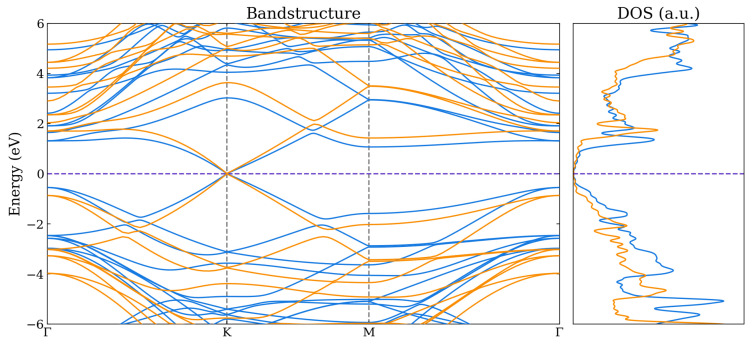
Band structure and total DOS for Graphene–B_4_C_3_ as calculated using the PBE (blue) and HSE06 (orange) functionals. The Fermi level is indicated by the purple dashed line.

For the bilayer systems, the band structures and total density of states (DOS) are shown in Figure 5, Figure 6 and Figure 7 as calculated using the HSE06 and PBE functionals. The corresponding atom-projected (or partial) PDOS are presented in Figures S9–S11. The band structures for the other lateral positions considered for the Graphene–Borophene and Graphene–B_4_C_3_ systems are shown in Figures S7 and S8, where there is a small difference between them.

The Graphene–Borophene system exhibits metallic behaviour due to the small occupancy of states at the K-point which are mainly carbon related as seen from the PDOS in Figure S9. Graphene–B_4_C_3_ appears to have kept the character of the respective monolayers, displaying essentially semi-metal behaviour with the B_4_C_3_ states located away from the Fermi level (i.e., above ≈1.2 eV and below ≈−0.8 eV) as seen from the PDOS in Figure S10. It is notable that the Graphene–BC_3_ heterojunction exhibits a sizeable density-of-states just above the Fermi level (see Figure S11) which is related to the relatively flat band near the M-point and along *K* to Γ in the Brillouin zone. For these bilayer systems, the main difference between the PBE and HSE06 appears to be the raising and lowering of conduction and valence bands of the HSE06, respectively, relative to those obtained by the PBE functional. At the K-point the graphene bands exhibit a very small band-gap opening of 20 meV and 50 meV for Graphene–B_4_C_3_ and Graphene–BC_3_, respectively.

#### 3.2.4. Schottky Barrier Height

**Figure 8:**
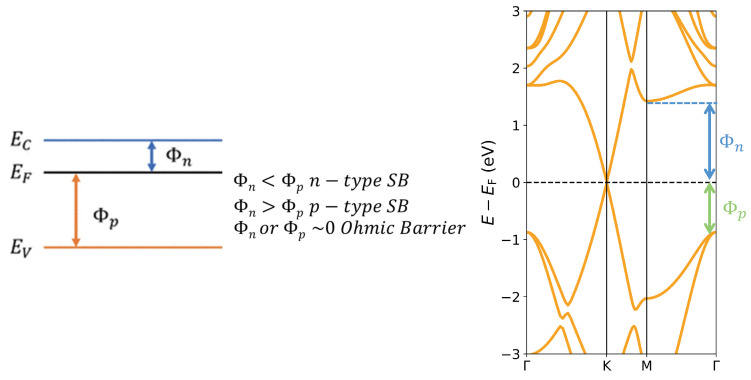
**Left**: schematic of the Schottky-Mott model showing the valence and conduction band energies, the Fermi energy, and *n*-type and *p*-type Schottky barriers labelled $E_{V}$, $E_{C}$, $E_{F}$, $\Phi_{n}$, and $\Phi_{p}$, respectively. **Right**: Example for the HSE06 calculated Graphene–B_4_C_3_ heterojunction showing the determined Schottky barrier height from the band structure.

The graphene/boron-carbide bilayer structures can function as a Schottky diode. A high Schottky barrier will block the reverse (metal-to-semiconductor) current, realizing the rectification effect of a diode. Based on the Schottky–Mott model (shown in Figure 8, left), at the metal–semiconductor interface [45] in *n*-type materials the Schottky barrier ($\Phi_{n}$) is defined as the energy difference between the Fermi level ($E_{F}$) and the conduction band minimum ($E_{C}$), that is, $\Phi_{n}=E_{C}-E_{F}$. Similarly, in *p*-type materials the Schottky barrier ($\Phi_{p}$) is defined as the energy difference between the Fermi level ($E_{F}$) and the VBM ($E_{V}$), that is, $\Phi_{p}=E_{F}-E_{V}$ [46,47]. The sum of two types of Schottky barriers is approximately equal to the band gap value ($E_{g}$) of the semiconductor, that is, $\Phi_{n}+\Phi_{p}=E_{g}$. For the Graphene–B_4_C_3_ system as illustrated in Figure 8, right, we find $\Phi_{n}=1.42$ eV (1.07 eV) for the HSE06 (PBE) and $\Phi_{p}=0.87$ eV (0.55 eV) for the HSE06 (PBE) signifying the heterojunction forms a *p*-type Schottky barrier since $\Phi_{p}<\Phi_{n}$. For the Graphene–BC_3_ heterostructure bilayer, we find $\Phi_{n}=0.19$ eV (0.003 eV) for the HSE06 (PBE) and $\Phi_{p}=1.42$ eV (0.39 eV) for the HSE06 (PBE) signifying an *n*-type Schottky barrier. For device applications a smaller Schottky barrier height or even an Ohmic contact is preferred to reduce contact resistance.

### 3.3. Optical Properties

Having determined the atomic and electronic properties of the bilayer systems, the optical properties are now investigated. In particular, the real part ($\epsilon_{1}$) and imaginary part ($\epsilon_{2}$) of the dielectric function are calculated, and from them the other optical properties can be determined (cf. Equations (4)–(8)). $\epsilon_{1}$ is a measure of the strength of the dynamical screening effects arising from charge excitations, while $\epsilon_{2}$ is a measure of light absorption as a consequence of neutral and plasmonic charge excitations. A negative value of the real part of the dielectric function indicates metallic character for the corresponding ranges of the electromagnetic spectrum.

It is first important to establish the appropriate **k**-point set to yield converged results. For graphene, we tested a number of **k**-point meshes, and found that 129×129×1 yielded converged results as shown in Figure S12, in good agreement with previous results [48]. We therefore used this **k**-point sampling density for the other single monolayers (whose unit cells are (2×2) relative to graphene), namely a 65×65×1 **k**-point set. The dielectric function for all the monolayers is shown in Figure S13 where the results are consistent with previous calculations, namely: graphene [48], BC_3_ [14,49], B_4_C_3_ [42] and borophene [50,51]. The response of the structures to light is highly dependent on the polarisation direction of incoming light, as seen by the anisotropic character of the in- and out-of-plane directions of the dielectric function. For in-plane, the well-known π plasmon of graphene can be seen emerging around 4 eV and the π−σ plasmon emerges at around 14 eV. The other monolayer systems also have a π-like plasmon which emerges for B_4_C_3_ around 3 eV, for BC_3_ around 2 eV and borophene around 1 eV. It can furthermore be seen from Figure S13 for the real part of the dielectric function in the out-of-plane direction for borophene, that a plasmon emerges at 9.8 eV as evidenced by the negative value which becomes positive around 10.5 eV.

As previously reported, the latter three monolayers, remarkably, exhibit visible light absorption which is also indicated by the in-plane imaginary part of the dielectric function seen in Figure S13. The static dielectric constants (real part of the dielectric constant at zero energy) for BC_3_ are 4.0 eV for in-plane polarization and 1.4 eV for out-of-plane polarization as obtained by the PBE. These values can be compared to 4.86 eV for in-plane polarization and 1.59 eV for out of plane polarization as obtained by Ref. [49] using the PBE functional. For B_4_C_3_ the in-plane static dielectric constant is 3.9 eV and the out-of-plane is calculated to be 1.5 eV. We note these values reflect the combined monolayer-vacuum system. In Figure S14 the corresponding optical properties are shown for the monolayer systems.

Before we present the results for the three heterostructure bilayer systems, we first perform a convergence check for the number of unoccupied bands to include in the calculation of the dielectric function. Figure S15 shows the results for the representative system Graphene–B_4_C_3_. It can be seen that 128 bands yield well-converged results and we hereafter use this value for all the bilayer calculations. Further tests of the energy convergence criteria are reported in Figure S16.

**Figure 9:**
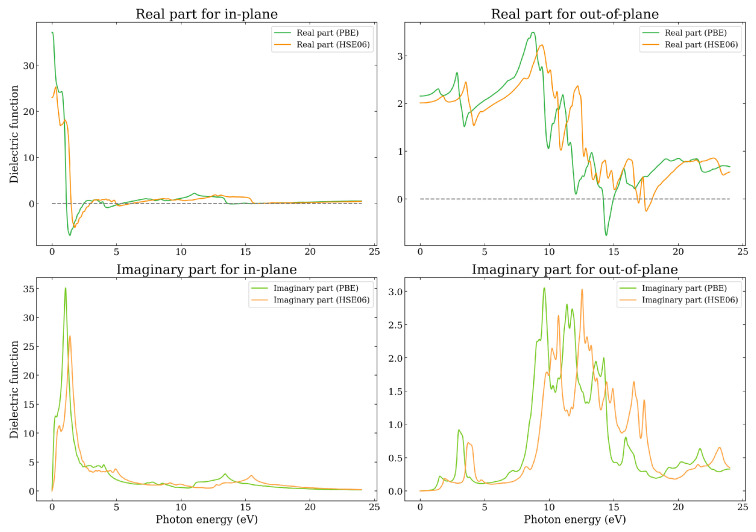
The real and imaginary parts of the in-plane and out-of-plane dielectric function as a function of photon energy as calculated using the PBE and HSE06 functionals for the Graphene–Borophene heterostructure.

The results for the dielectric function of the Graphene–Borophene heterojunction are shown in Figure 9 as obtained using both the PBE and HSE06 functionals. It can firstly be seen that, in comparison to the PBE results, the HSE06 yields a blue shift of the features to higher energies. The real part exhibits characteristics of both graphene and borophene with the presence of plasmons emerging around the same energies of the respective monolayers (i.e., 5 eV (4 eV), 2 eV (1 eV) for HSE06 (PBE) respectively). There is, however, a significant difference in the out-of-plane dielectric function compared to the monolayer systems. Firstly, it can be noticed that the borophene related plasmon that emerges at around 9.8 eV in the monolayer, has shifted to higher energies and now appears at around 17 eV (HSE06) 14 eV (PBE) with a plasmon resonance frequency at about 18 eV (HSE06) 15 eV (PBE), as determined by the energy at which the real part of the dielectric function changes from negative to positive. Also, a new feature with two peaks occurs around 2–5 eV (1–4 eV) for the HSE06 (PBE) for both the real and imaginary parts of the dielectric function. This feature in the imaginary part is attributed to interband transitions involving B- and G-derived states about the K-point and along the direction towards the M- and Γ-points—a similar feature appears for the other bilayer heterojunctions, albeit showing just one main peak. For in-plane polarization, the first main peak of $\epsilon_{2}$ happens in the visible range, and is related to $\pi -\pi^{*}$ transitions. The main peaks along the out-of-plane direction are broad and occur in the energy range between 9–18.5 eV (8–17.5 eV) for the HSE06 (PBE), being related to $\pi -\sigma^{*}$ and $\pi^{*}-\sigma$ transitions.

**Figure 10:**
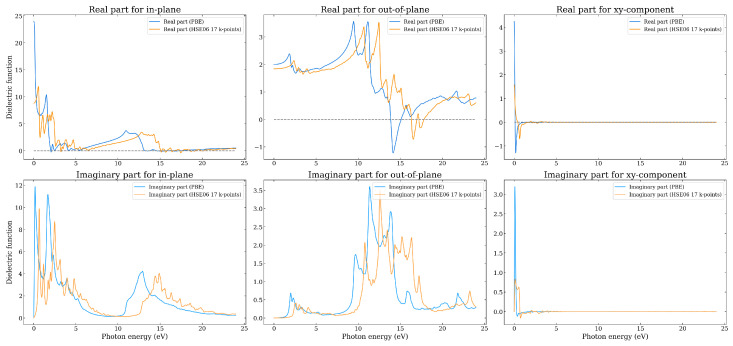
The real and imaginary parts of the in-plane and out-of-plane dielectric function as a function of photon energy as calculated using the PBE and HSE06 functionals for the Graphene–BC_3_ heterostructure.

In Figure 10 the results for the dielectric function of the Graphene–BC_3_ heterostructure are shown, as obtained using both the PBE and HSE06. In contrast to the Graphene–Borophene system, the real part of the dielectric function is positive for all the energy range considered. There are however energies at which it goes to zero, namely in the region 2.5–5 eV and from 13 eV (15 eV) onwards for the PBE (HSE06). When $\epsilon_{1}$ is zero but not negative, it suggests the system is close to the resonance but has not fully entered the regime where strong optical reflection or plasmonic modes are dominant, as those would typically require $\epsilon_{1}<0$. Also different to the Graphene–Borophene system is the sizeable in-plane value of both $\epsilon_{1}$ and $\epsilon_{2}$ in the region 10–15 eV. Similar to the Graphene–Borophene system, however, is the negative out-of-plane real part of the dielectric function at around 16–17 eV (14–15 eV) from HSE06 (PBE) indicating the presence of a plasmon. Interestingly, monolayer BC_3_ does not exhibit this feature (unlike borophene), and it is thus presumably related to the π−σ-type plasmon present for graphene at ≈15 eV but which involves transitions between states involving both monolayers. For in-plane polarization, the first main peaks of $\epsilon_{2}$ occur in the visible range, and are related to $\pi -\pi^{*}$ transitions. The main peaks along the out-of-plane direction are broad and are in the energy range between 11–17 eV (9–16 eV) for the HSE06 (PBE), being related to $\pi -\sigma^{*}$ and $\pi^{*}-\sigma$ transitions.

Interestingly, and in contrast to Graphene–Borophene, the dielectric tensor for this system contains complex non-zero off-diagonal components, as seen in Figure 10 for the xy component. Figure S17 shows all of the components, in which it is observed that components xy=−yx,xz=−zx,yz=−zy. The dielectric tensor describes the response of a material to an external electric field, that is, it characterizes how the polarization of the material changes in response to the applied electric field. For an incident photon, the dielectric tensor components describe the response of the material to the photon’s electric field component along different directions, e.g., the element $\epsilon_{xy}$ and $\epsilon_{yx}$ describe the response to an electric field applied along the *x*-direction, resulting in polarization along the *y*-direction and vice versa. It is clear that the components are however non-zero only at very low energy and are small in value.

**Figure 11:**
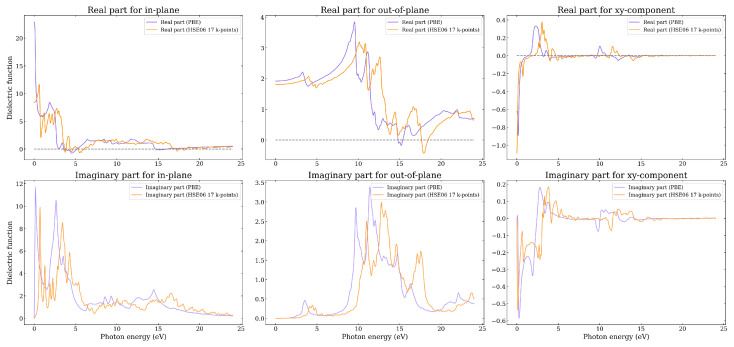
The real and imaginary parts of the in-plane and out-of-plane dielectric function as a function of photon energy as calculated using the PBE and HSE06 functionals for the Graphene–B_4_C_3_ heterostructure.

In Figure 11 the results for the dielectric function of the Graphene–B_4_C_3_ heterostructure are shown, as obtained using both the PBE and HSE06. It can be seen that the real part of the dielectric function for the in-plane component is negative in the region 5–6 eV HSE06 (4–5 eV PBE) and 17–18 eV HSE06 (15–16 PBE) thus hosting plasmons in the ultraviolet. The out-of-plane component also displays negative values about 17–18 eV HSE06 (14.5–15.5 eV PBE). Similarly to Graphene–B_4_C_3_ for in-plane polarization, the first main peaks of $\epsilon_{2}$ are also in the visible range, and the main peaks along the out-of-plane direction are broad and occur in the energy range between 10–18 eV (9–17 eV) for the HSE06 (PBE).

Similar to Graphene–BC_3_, the dielectric tensor for this system contains complex non-zero off-diagonal components, as seen in Figure 11. Figure S18 shows all of the components where xy=−yx,xz=−zx,yz=−zy. In this case the dielectric function is non-zero for a considerable range of energies, i.e., from 0–5 eV and from 10–15 eV. The Graphene–B_4_C_3_ system has a P3 space group which has 3-fold rotational symmetry along the *c* (*z*-coordiante)-axis and it lacks mirror symmetry planes perpendicular to this axis or within the a,b (x,y plane). This lower symmetry allows for the presence of off-diagonal elements in the dielectric tensor. This means that the dielectric response of the material with this space group can exhibit anisotropic coupling between different crystallographic directions. The presence of complex off-diagonal elements in the dielectric tensor of a material with the P3 space group indicates that the material has anisotropic and possibly chiral optical properties, which could lead to effects like optical activity. It furthermore indicates Graphene–B_4_C_3_ dissipates energy differently depending on the direction of the electric field.

**Figure 12:**
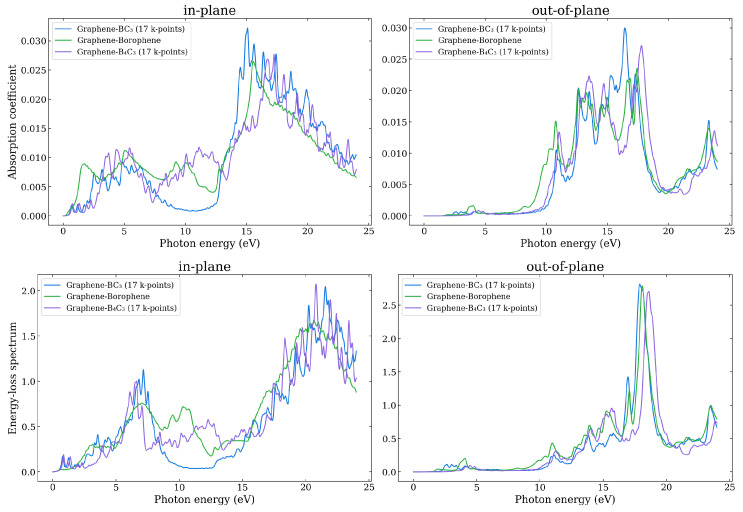
The in-plane and out-of-plane adsorption coefficient (upper) and energy loss spectrum (lower) as a function of photon energy as calculated using the HSE06 functional.

In order to further explore the optical properties of the heterostructure bilayers, the absorption coefficient, loss function, reflectivity, refractive index, and extinction coefficient are calculated. Figure 12 shows the absorbance and loss function for the three systems for in-plane and out-of-plane polarizations. In Figures S19–S23 all the components are shown. The absorbance threshold corresponds to where the imaginary part of the dielectric function, $\epsilon_{2}\left(\omega \right)$, shows its first peak. For all systems the adsorption coefficient starts very low and increases with energy, consistent with the lowest direct transition energies in the band structure. It is clear that there is absorption in the visible region of the spectrum (1.7–3.3 eV). It is noticeable that the Graphene–Borophene heterostructure has a significantly greater absorbance than the other two systems where it rises sharply around 1 eV. This can be attributed to transitions between the flat region of valence band about the Γ point and the low lying conduction band in the same region of the Brillouin zone. The Graphene–BC_3_ heterostructure exhibits a similar sharp rise beginning at a slightly higher energy of around 2.5 eV, followed by Graphene–B_4_C_3_ at around 3.5 eV due to transitions between the relatively flat valence and conduction bands along the Γ-M direction.

The loss function in Figure 12 presents a sharp feature at low energy, related to Dirac-like plasmons, for the Graphene–BC_3_ and Graphene–B_4_C_3_ structures which is absent for the Graphene–Borophene bilayer. The broader features from 3–8 eV can be attributed to π-like plasmons. We note that individual interband transitions presumably coexist with the plasmons forming a broad background. The coexistence of individual transitions and plasmons in the same energy range lead to a coupling of the two types of excitations, and hence finite plasmon lifetimes as well as interband transitions [52]. The features in the loss function for energies around 15 eV where there is onset of a sharp increase may be associated with π−σ-type plasmons, where for all three bilayer heterostructures there is a plasmon supported at around this energy (as indicated by the negative real part of the out-of-plane component of the dielectric function).

**Figure 13:**
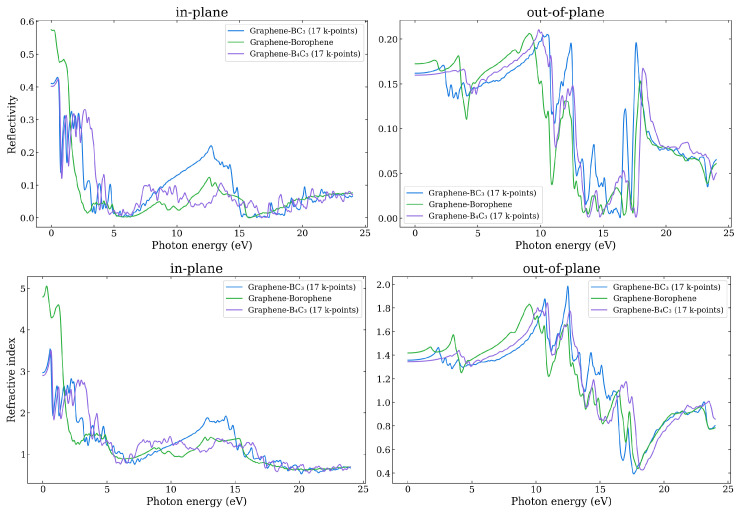
The in-plane and out-of-plane reflectivity (upper) and refractive index (lower) as a function of photon energy as calculated using the HSE06 functional.

In Figure 13 the reflectivity and refractive index are shown for the three heterostructures, as obtained using the HSE06, for polarization in the in-plane and out-of-plane directions. All of the components for each heterostructure are shown in Figures S25–S27. The refractive index is related to the real and imaginary parts of the dielectric constant. It can be seen that the trends in refractive index are similar to $\epsilon_{1}$, which indicates that the effect of the real part of the dielectric function on the refractive index plays the leading role. The refractive index determines how much the path of light is bent, or refracted, when entering a material. For visible light, most transparent materials have refractive indices between 1 and 2. The absolute refractive index of an optical medium is defined as the ratio of the speed of light in vacuum and the phase velocity (speed at which the crests of the wave move) of light in a material. Given that it is possible for the phase velocity to travel faster than the speed of light in vacuum, the refractive index can be less than 1. For frequencies near the plasmon resonance in 2D materials, the refractive index can drop below one due to the strong interaction between light and the plasmonic oscillations.

It can be seen that the refractive index for in-plane polarization is close to, and less than one for energies in the range of ∼5–7 eV and greater than ∼17 eV for all three systems, while the out-of-plane refractive index is close to or less than one for energies greater than about 14 eV. This means that in these ranges of the electromagnetic spectrum, the structures are highly transparent, and it is in the region of where the plasmons occur.

The extinction coefficient (see Figure S23) for out-of-plane polarization displays a broad maximum from zero up to around 13 eV, while the in-plane polarization exhibits a maximum value in the region from 0–2, 0–2.5 and 0–3 eV, for the Graphene–Borophene, Graphene–BC_3_ and Graphene–B_4_C_3_ heterostructures, respectively. There is also a broad peak in the region of around 7–15 eV. This means that in these energy regions, photons will be absorbed very fast.

## 4. Conclusions

In the present work, the atomic, electronic, and optical properties of Graphene–Borophene, Graphene–BC_3_ and Graphene–B_4_C_3_ bilayer heterostructure systems have been investigated using first-principles calculations employing the HSE06 and PBE functionals. We find all systems have a small binding energy between the layers showing that graphene interacts weakly with the monolayers via the van der Waals interaction. The Graphene–Borophene bilayer is found to be metallic, while Graphene–BC_3_ and Graphene–B_4_C_3_ structures maintain the characteristic linear bands of Graphene about the K-point with only very small band gap opening (20–50 meV). The calculated optical properties show absorbance in the visible region of the spectrum for all heterostructures with Graphene–Borophene exhibiting the strongest absorption. All heterostructures support plasmons with Graphene–Borophene exhibiting two at low energy, one emerging at 2 eV and the other around 5 eV. The Graphene–B_4_C_3_ bilayer heterostructure is found to have non-negligible off-diagonal elements in the dielectric tensor over a range of energies which suggests it could exhibit interesting optically active (chiral) effects.

## Figures and Tables

**Table 1:** Optimized lattice constants, inter-atomic distances, and band gaps of monolayer BC_3_, borophene, B_4_C_3_, and graphene. Here, *a* is the lattice parameter with a=b for all systems, B-B, C-C, and B-C are the boron-boron, carbon-carbon, and boron-carbon bond lengths. Borophene and B_4_C_3_ have B-B and B-C bonds of different lengths, these are identified by labels $l_{1}$ … $l_{7}$ in Figure 1. The band gap $E_{g}$ is calculated using the PBE and HSE06 exchange-correlation functionals, values in brackets are from previous theoretical studies. “Nature” indicates the electronic character of the system with “indirect” referring to an indirect semiconductor.

System	*a* (a=b) (Å)	B-B (Å)	C-C (Å)	B-C (Å)	$E_{g-\mathrm{PBE}}$ (eV)	$E_{g-\mathrm{HSE}06}$ (eV)	Nature
BC_3_	5.169	-	1.421	1.563	0.634 (0.62–0.66) [38,40,41]	1.822 (1.83) [41]	indirect
Borophene	5.051	1.689 ($l_{1}$) 1.672 ($l_{2}$) 1.707 ($l_{3}$)	-	-	-	-	metallic
B_4_C_3_	4.690	1.689 ($l_{4}$)	-	1.592 ($l_{5}$) 1.517 ($l_{6}$) 1.548 ($l_{7}$)	1.647	2.381 (2.39) [42]	indirect
Graphene	2.467	-	1.424	-	-	-	semimetal

**Table 2:** Optimized structural properties for each bilayer heterojunction system. Namely, the lowest energy lateral coordination (or position) of the stacking relative to graphene, the optimized lattice constant *a*, strain relative to the respective monolayer, $S_{G}$ and $S_{\mathrm{BC}/B}$, inter-planar distance $d_{\mathrm{IL}}$ and electronic nature. A negative and positive value of the percentage strain corresponds to a compressive and tensile strain, respectively.

System	Coordination	*a* (a=b) (Å)	$S_{G}$	$S_{\mathrm{BC}/B}$	$d_{\mathrm{IL}}$ (Å)	Nature
Graphene–BC_3_	Hollow	5.044	2.23%	−2.42%	3.372	semi-metal
Graphene–Borophene	Top	4.979	0.91%	−1.43%	3.505	metallic
Graphene–B_4_C_3_	Top	4.846	−1.78%	3.33%	3.514	semi-metal

## Data Availability

The original data presented in the study are openly available in the GitHub repository Photonico/Graphene-BC_20230622 (accessed on 14 October 2024) at https://github.com/Photonico/Graphene-BC_20230622.

## Supplementary Materials

The following supporting information can be downloaded at: https://www.mdpi.com/article/10.3390/nano14201659/s1, Figure S1: **k**-point convergence; Figure S2: Monolayer lattice parameters; Figure S3: Heterostructure lattice paramaters; Table S1: Heterostructure lateral position tests; Figure S4: Graphene–BC_3_ interlayer distance; Figure S5: Graphene–Borophene interlayer distance; Figure S6: Graphene–B_4_C_3_ interlayer distance; Figure S7: Graphene–Borophene PBE band structure; Figure S8: Graphene–B_4_C_3_ PBE band structure; Figure S9: Graphene–Borophene projected density of states; Figure S10: Graphene–B_4_C_3_ projected density of states; Figure S11: Graphene–BC_3_ projected density of states; Figure S12: Dielectric function **k**-point convergence for graphene; Figure S13: Monolayer dielectric functions; Figure S14: Monolayer optical properties; Figure S15: Dielectric function band convergence; Figure S16: Dielectric function energy criteria convergence; Figure S17: Graphene–BC_3_ dielectric tensor components; Figure S18: Graphene–B_4_C_3_ dielectric tensor components; Figure S19: Heterostructure adsorption coefficient; Figure S20: Heterostructure loss function; Figure S21: Heterostructure reflectivity; Figure S22: Heterostructure refractive index; Figure S23: Heterostructure extinction coefficient.
