# Supplementary material for: Electronic and Optical Properties of 2D Heterostructure Bilayers of Graphene, Borophene and 2D Boron Carbides from First Principles
Source: Nanomaterials (Basel). 2024 Oct 16;14(20):1659. doi: 10.3390/nano14201659 (PMC11510170; doi:10.3390/nano14201659)
Supplement: Supplementary file 1 [file nanomaterials-14-01659-s001.zip › nanomaterials-3256082-supplementary.pdf]

Electronic and Optical Properties of 2D Heterostructure Bilayers of Graphene, Borophene and 2D Boron Carbides from First Principles – Supplementary Information

Lu Niu[1], Oliver J. Conquest[1*], Carla Verdi[1,2], and Catherine Stampfl[1*]

[1]School of Physics, The University of Sydney, Sydney 2006, NSW, Australia
[2]School of Mathematics and Physics, The University of Queensland, Brisbane, Queensland 4072, Australia
[*]Corresponding authors: oliver.conquest@sydney.edu.au, catherine.stampfl@sydney.edu.au

September 2024

The supplementary information below includes results on the determination of the lattice parameters used in the calculations for the graphene, borophene, BC_3 and B_4C_3 monolayers, and the associated heterostructure bilayer systems considered for various relative lateral displacements, as well as convergence tests of **k**-point sets and the band structure for the monolayers and all bilayer systems investigated, as calculated using the PBE and HSE06 functionals.

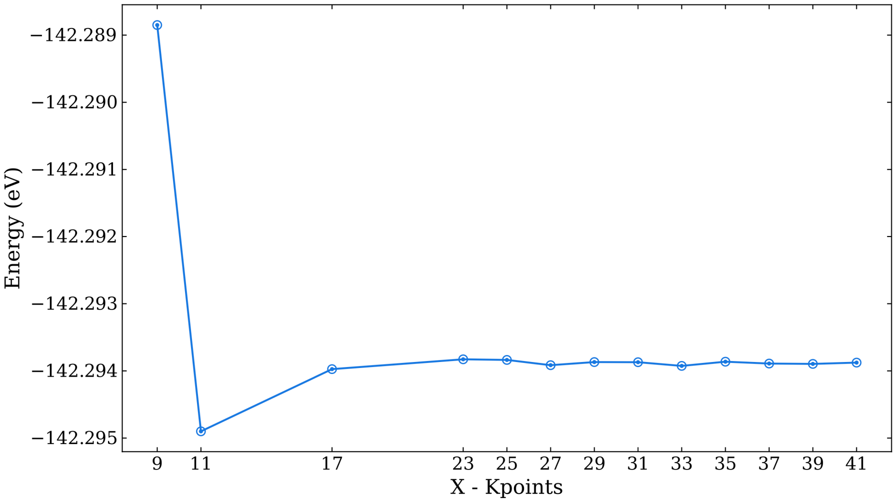

Figure S1: Total energy versus number of **k**-points in the sampling for the Brillouin zone integrations for the bilayer system Graphene−BC_3. "X" represents an X×X×1 **k**-point set.

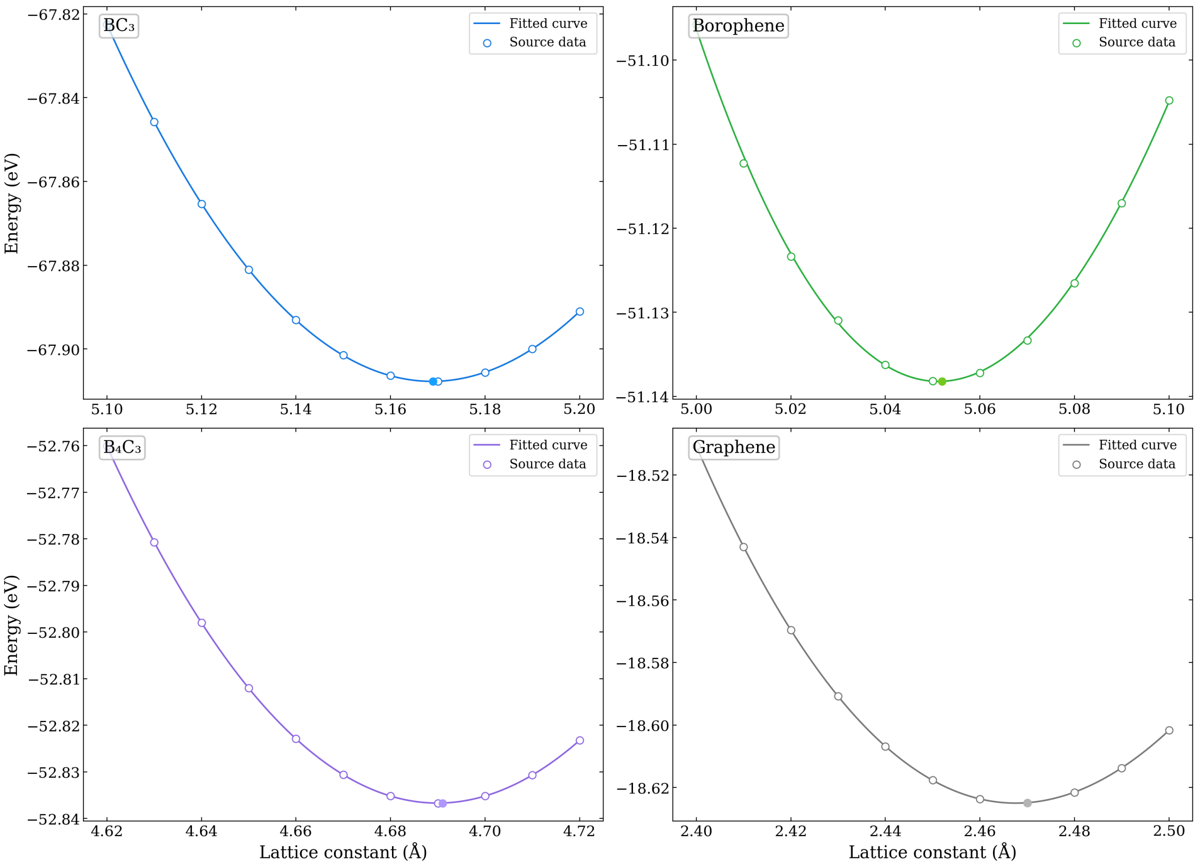

Figure S2: Total energy versus lattice constant for the four monolayer systems: Graphene, borophene, BC_3, and B_4C_3. The lattice constant yielding the lowest energy is shown by the full circle in each plot.

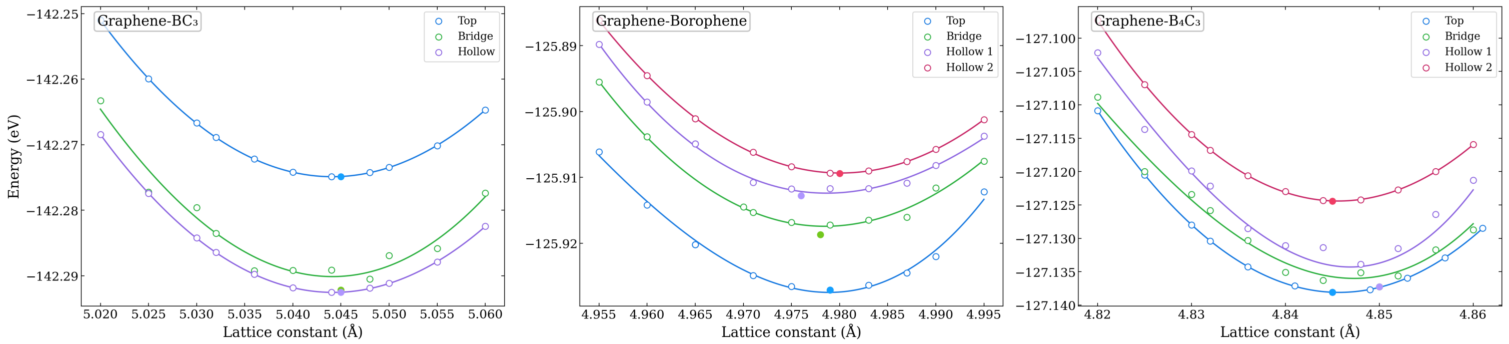

Figure S3: Total energy versus lattice constant for each bilayer system for the various stacking positions considered. The lines are fitted curves with the full circle indicating the lowest energy structure.

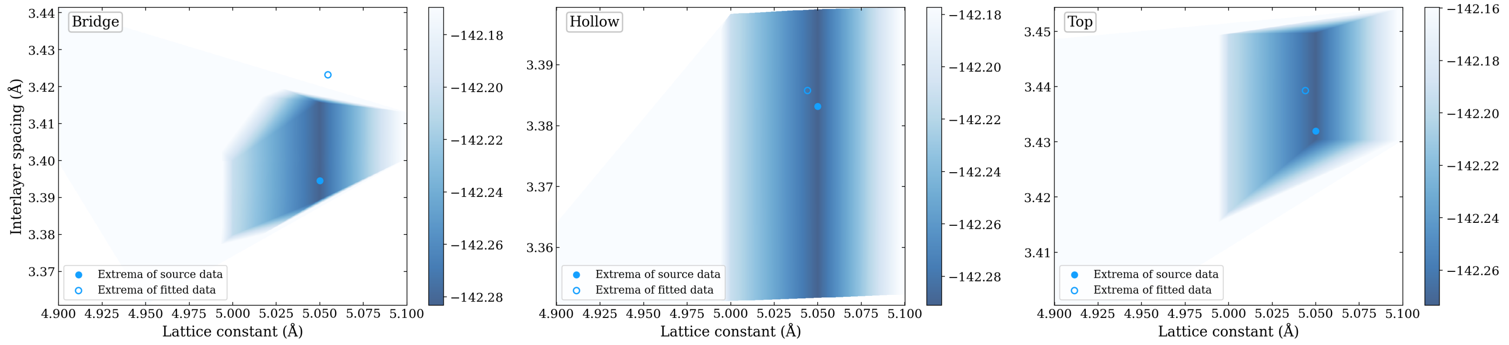

Figure S4: Total energy versus lattice constant and interlayer distance for the Graphene-BC_3 bilayer for the various lateral positions considered. The determined equilibrium geometry is indicated by the "Extrema of fitted data" result.

System	Site	Lattice constant (Å)	Inter-planar spacing (Å)	Total energy (eV)	Relative energy (eV)
G-BC_3	**Hollow**	**5.044**	**3.375**	**−142.293**	**0.000**
	Bridge	5.044	3.384	−142.290	0.002
	Top	5.044	3.432	−142.275	0.018
G-Borophene	**Top**	**4.979**	**3.496**	**−125.927**	**0.000**
	Bridge	4.979	3.505	−125.921	0.006
	Hollow 1	4.979	3.551	−125.914	0.013
	Hollow 2	4.979	3.548	−125.909	0.018
G-B_4C_3	**Top**	**4.846**	**3.514**	**−127.138**	**0.000**
	Bridge	4.846	3.536	−127.135	0.003
	Hollow 1	4.846	3.545	−127.133	0.005
	Hollow 2	4.846	3.562	−127.124	0.014

Table S1: For each bilayer system, for the various lateral positions (sites) considered, the optimized lattice constant, inter-layer spacing, total energy and relative energy are given. The relative energy is given with respect to the most favourable (lowest energy) structure for each bilayer system.

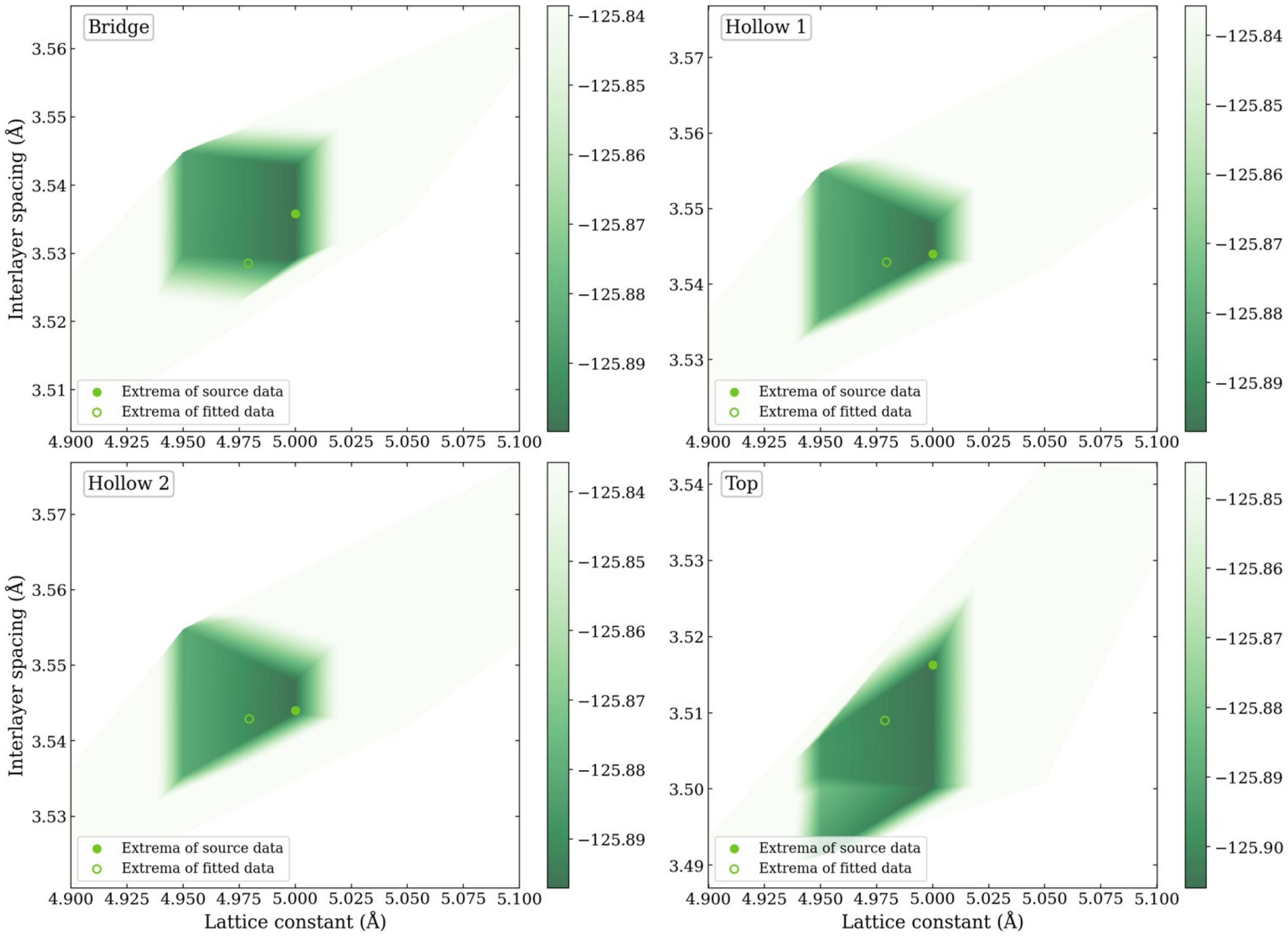

Figure S5: Total energy versus lattice constant and interlayer distance for the Graphene-Borophene bilayer for the various lateral positions considered. The determined equilibrium geometry is indicated by the ”Extrema of fitted data” result.

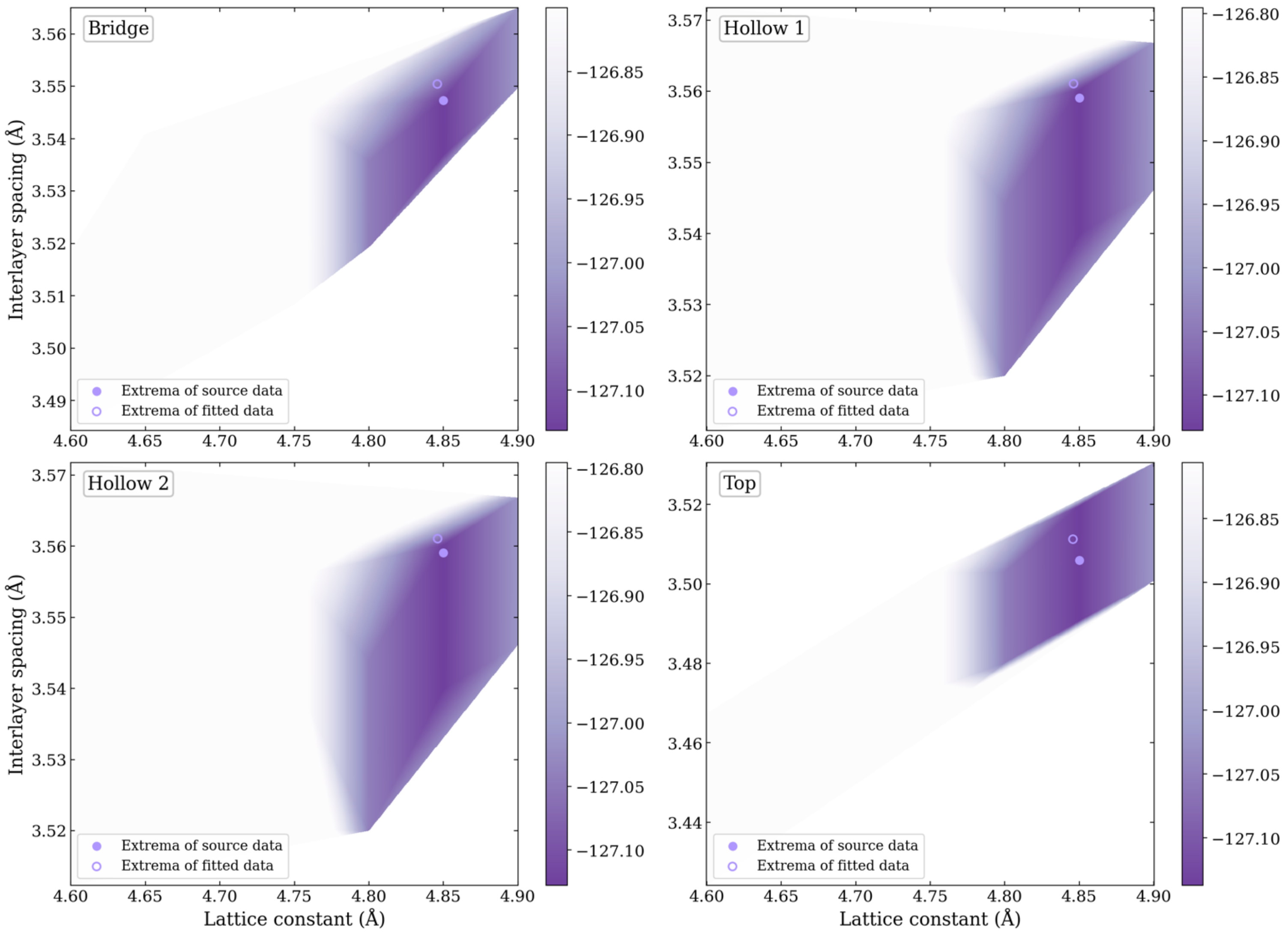

Figure S6: Total energy versus lattice constant and interlayer distance for the Graphene-B_4C_3 bilayer for the various lateral positions considered. The determined equilibrium geometry is indicated by the "Extrema of fitted data" result.

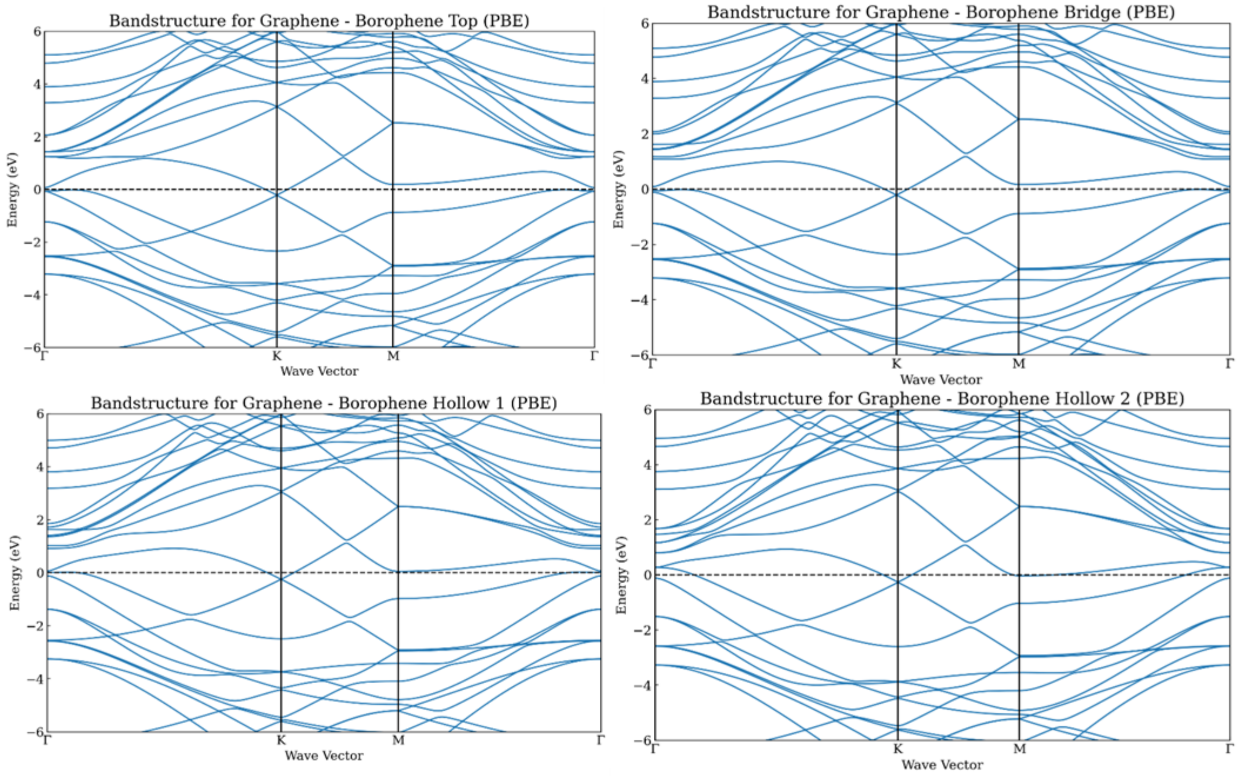

Figure S7: Band structure for the Graphene-Borophene bilayer as calculated using the PBE functional for the various lateral positions considered.

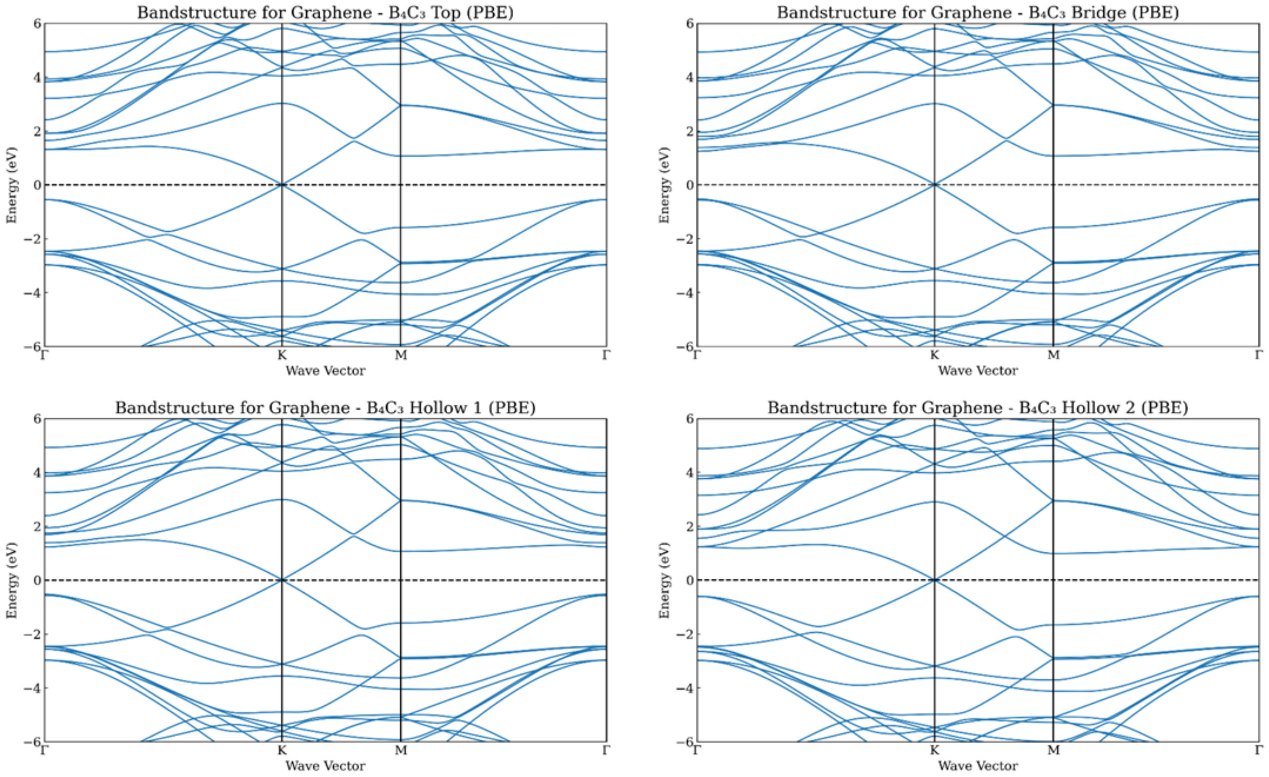

Figure S8: Band structure for the Graphene-B_4C_3 bilayer as calculated using the PBE functional for the various lateral positions considered.

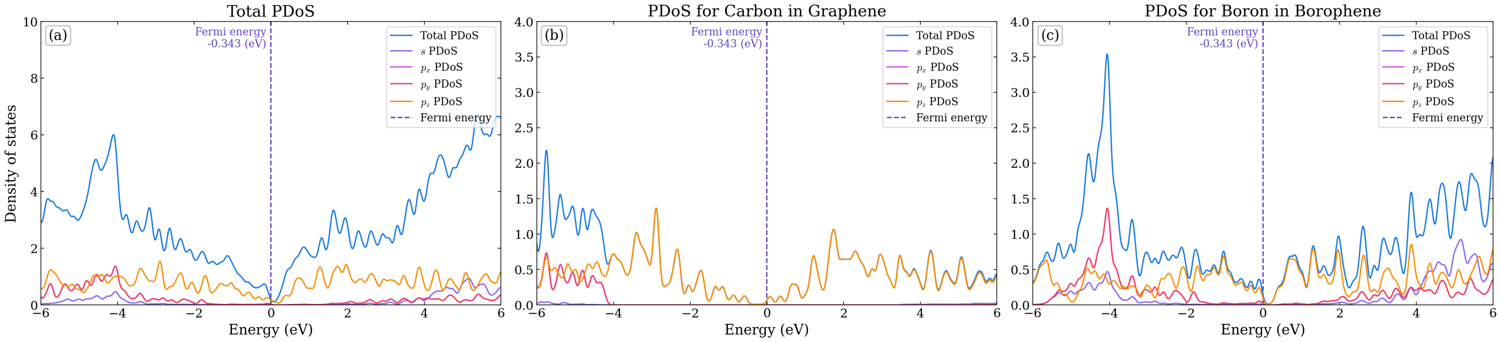

Figure S9: Projected density-of-states for the Graphene-Borophene heterostructure calculated using the HSE06 functional.

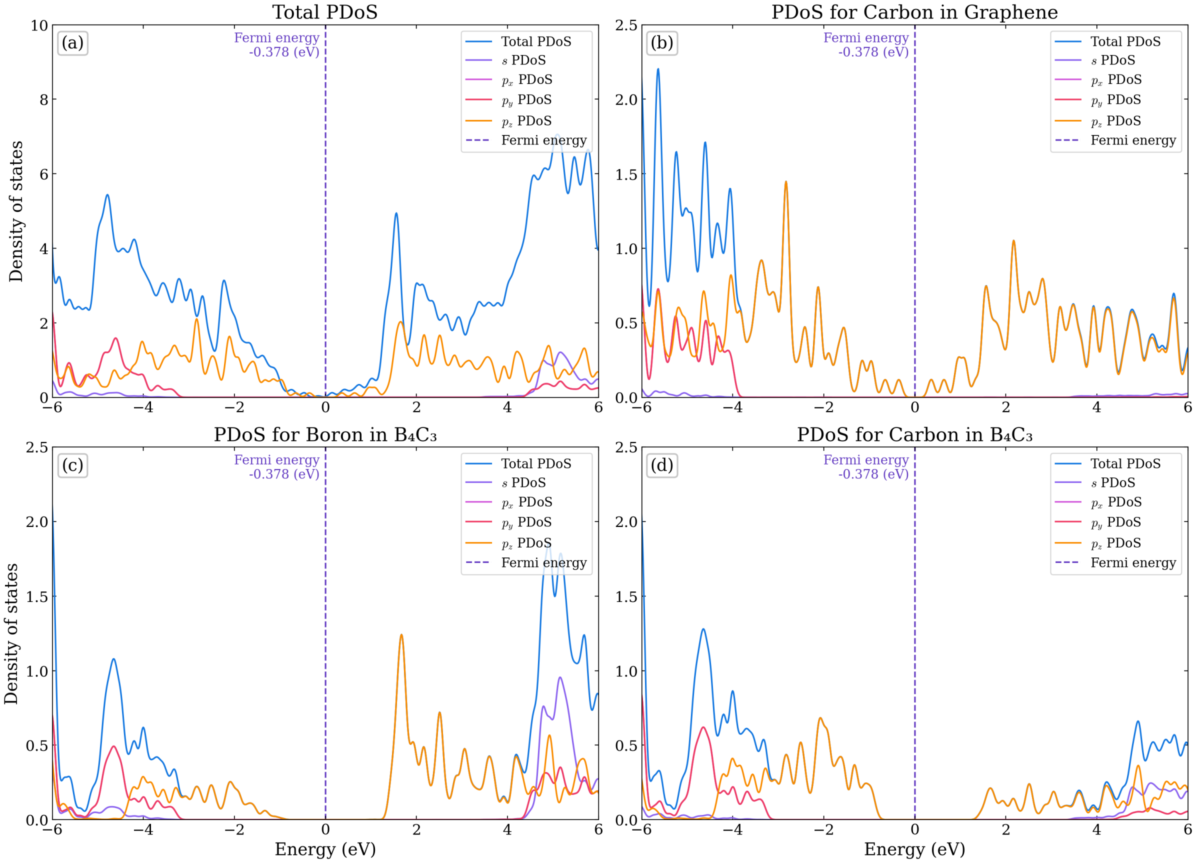

Figure S10: Projected density-of-states for the Graphene-B_4C_3 heterostructure calculated using the HSE06 functional.

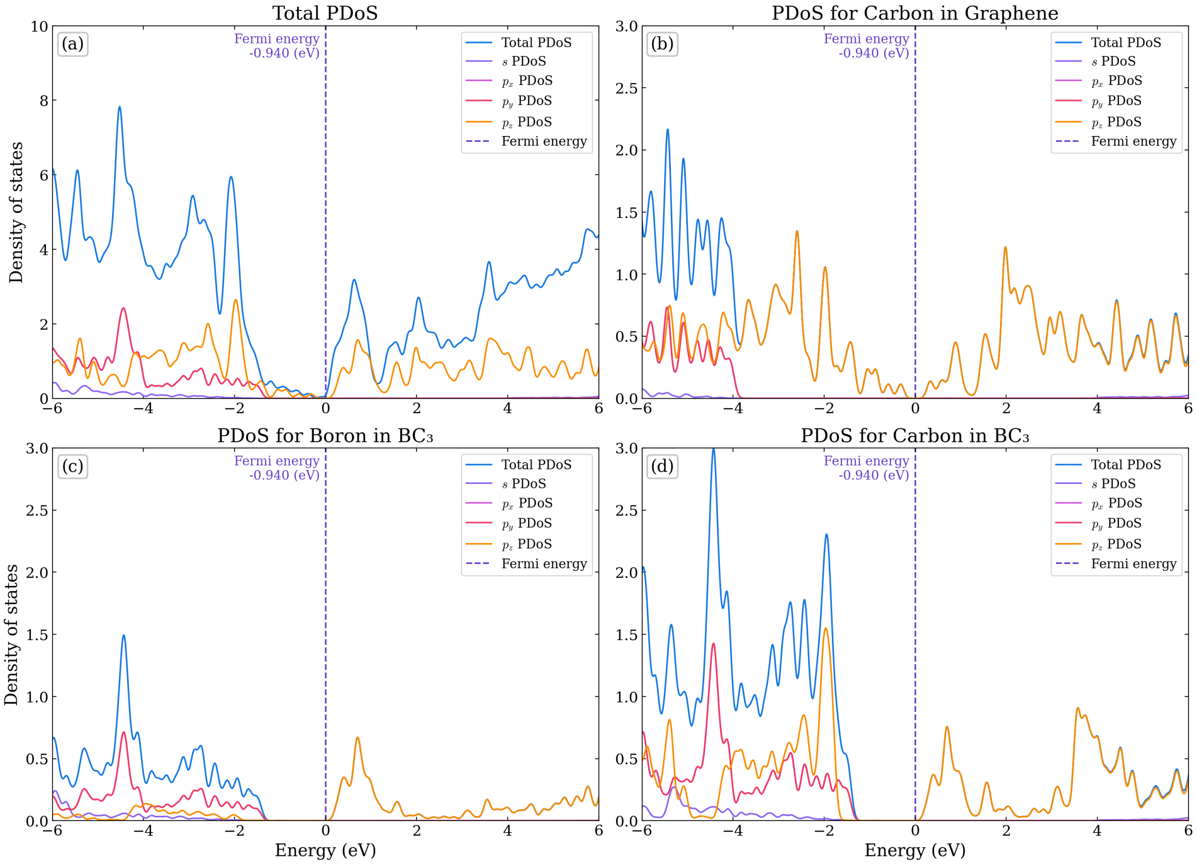

Figure S11: Projected density-of-states for the Graphene-BC_3 heterostructure calculated using the HSE06 functional.

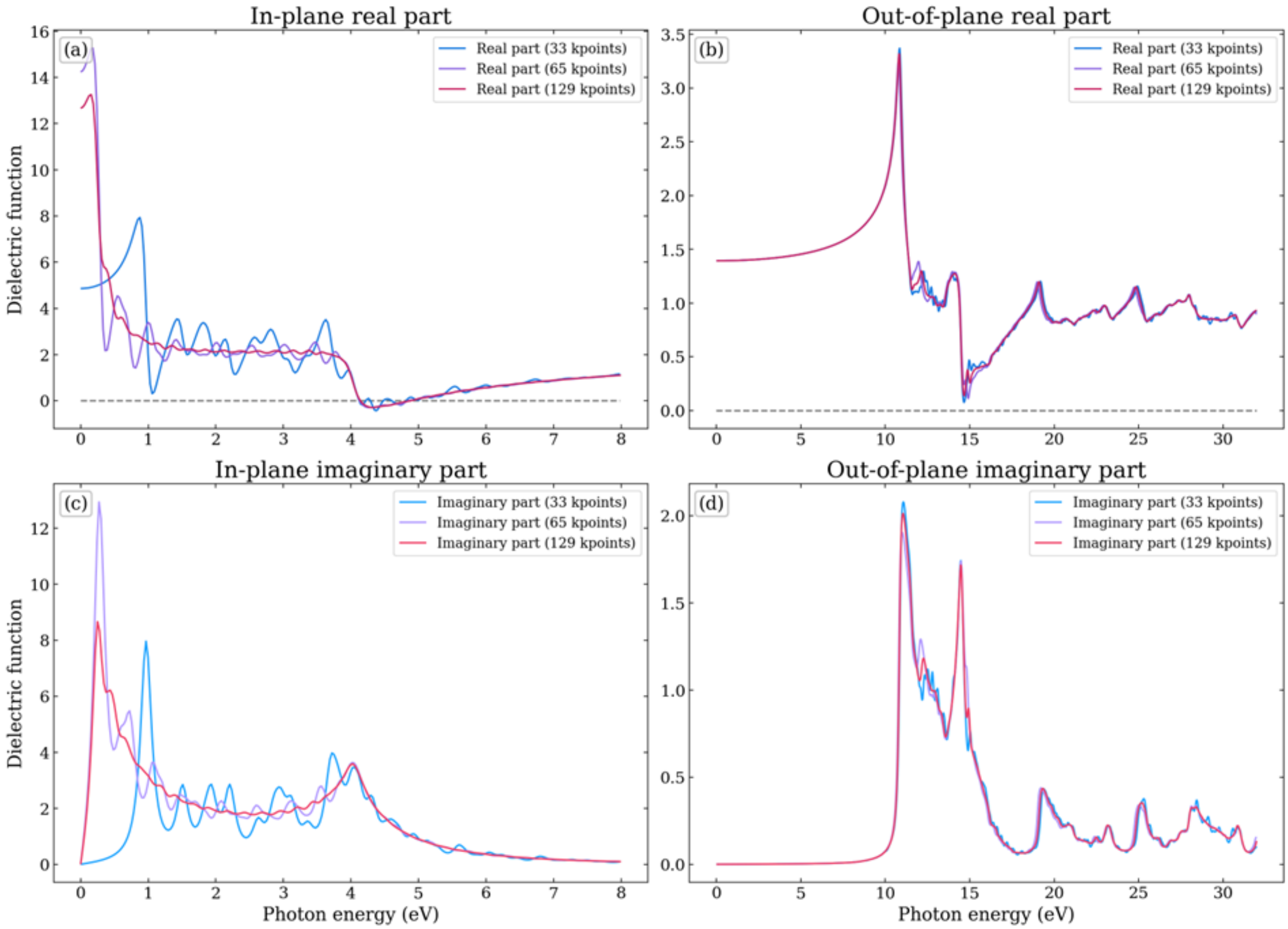

Figure S12: Dielectric function for graphene as calculated using the PBE functional for various **k**-point sets, where e.g. 33 kpoints means a 33×33×1 **k**-point mesh.

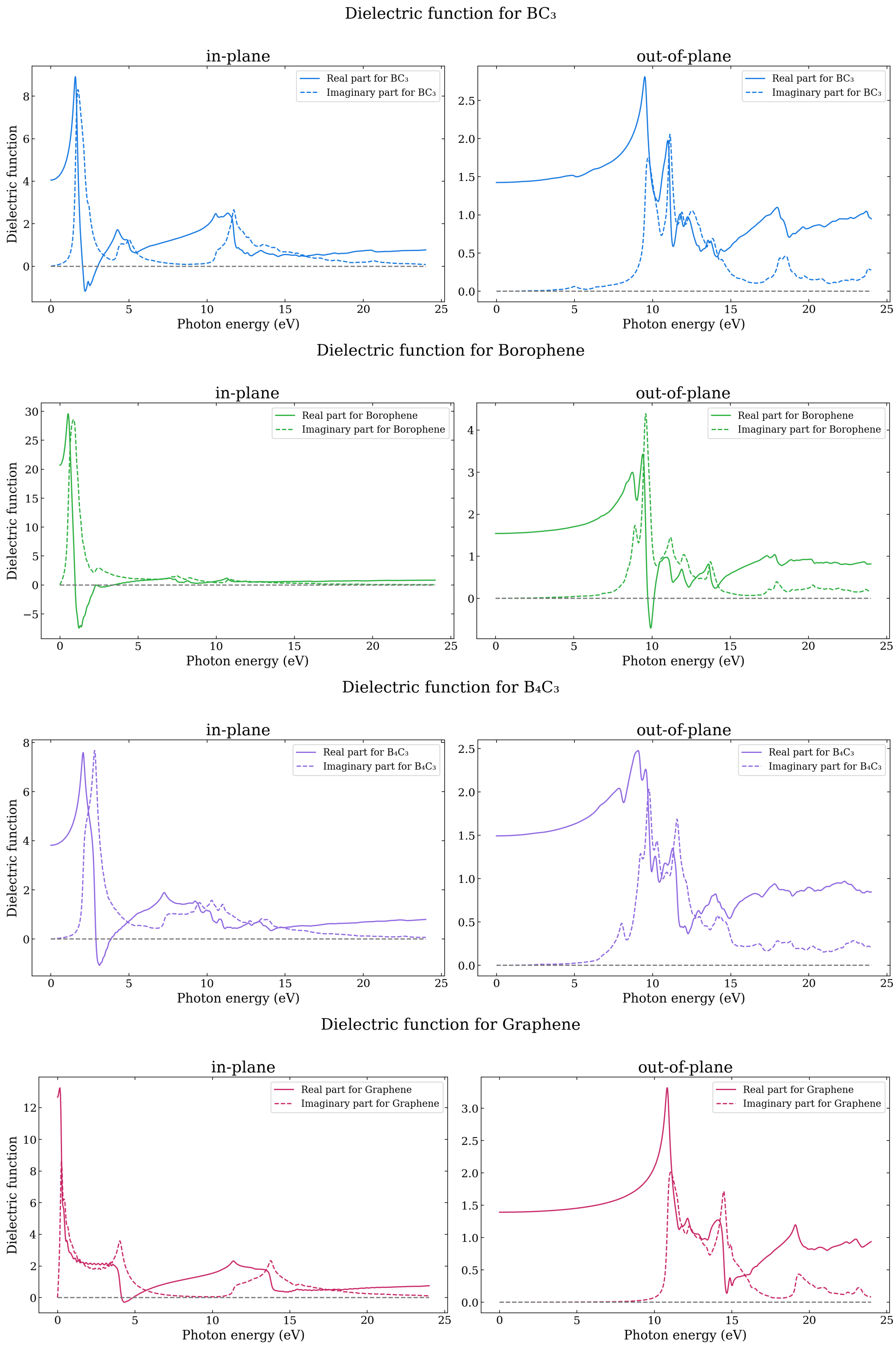

Figure S13: Real and imaginary part of the dielectric function for the monolayers as calculated using the PBE functional with a (129×129×1) **k**-point set for graphene and a (65×65×1) mesh for the other monolayers.

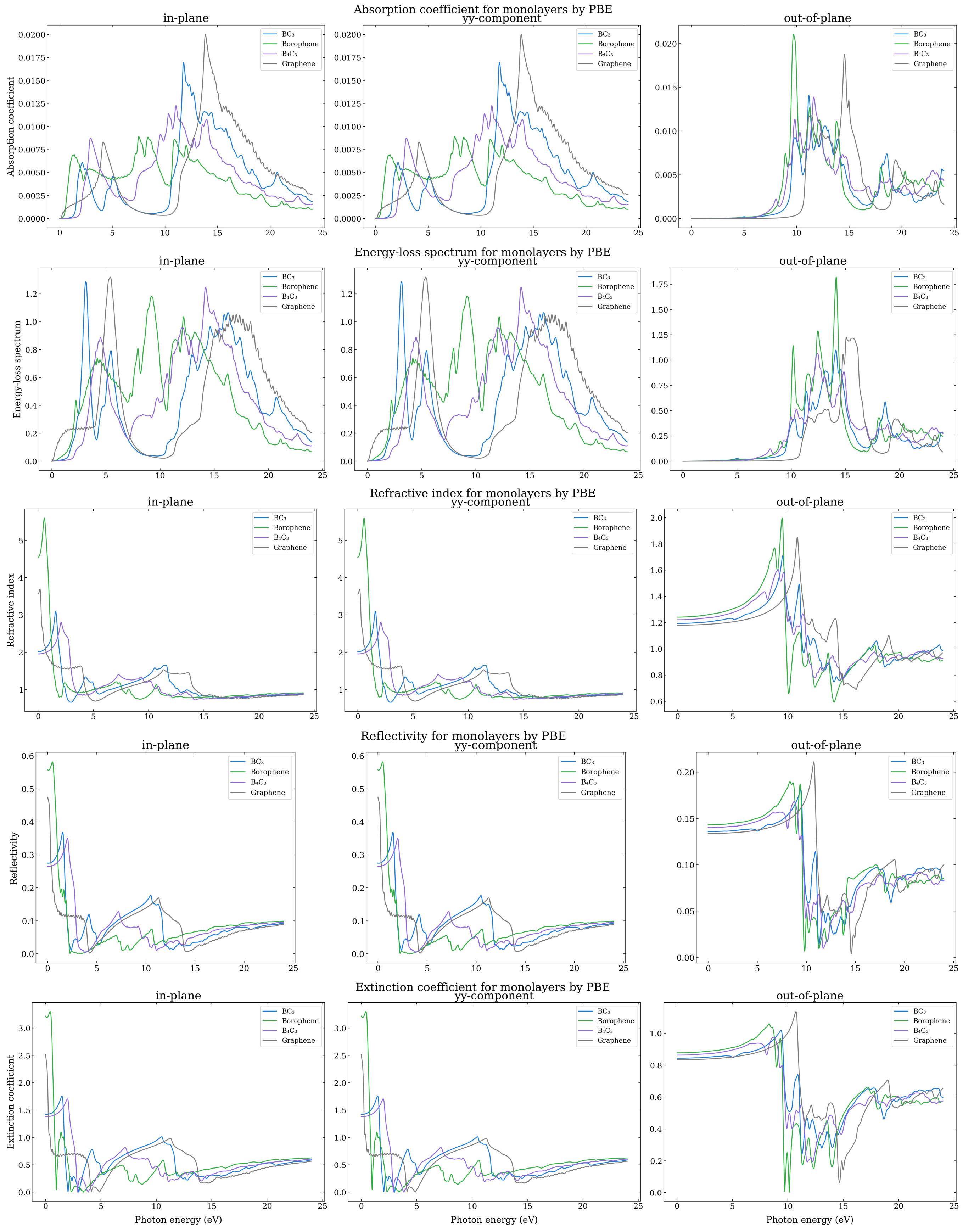

Figure S14: Optical properties for all the four monolayers as calculated using the PBE functional with a (129×129×1) k-point set for graphene and a (65×65×1) **k**-point set for the borophene and boron-carbide monolayers.

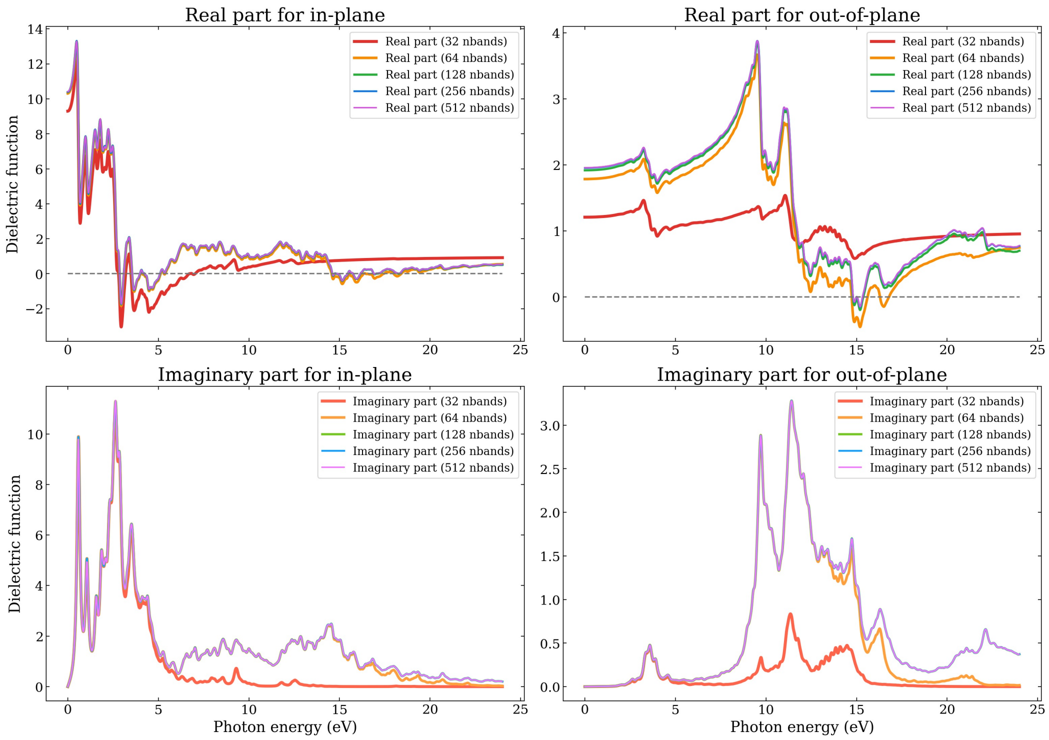

Figure S15: Convergence test for the number of unoccupied bands in calculating the dielectric function of the Graphene-B_4C_3 heterostructure bilayer as calculated using the PBE functional.

We also test the effect of the energy convergence criteria "EDIFF" on the calculated dielectric function in Figure S16 along with different **k**-point meshes and comparison of HSE06 and PBE.

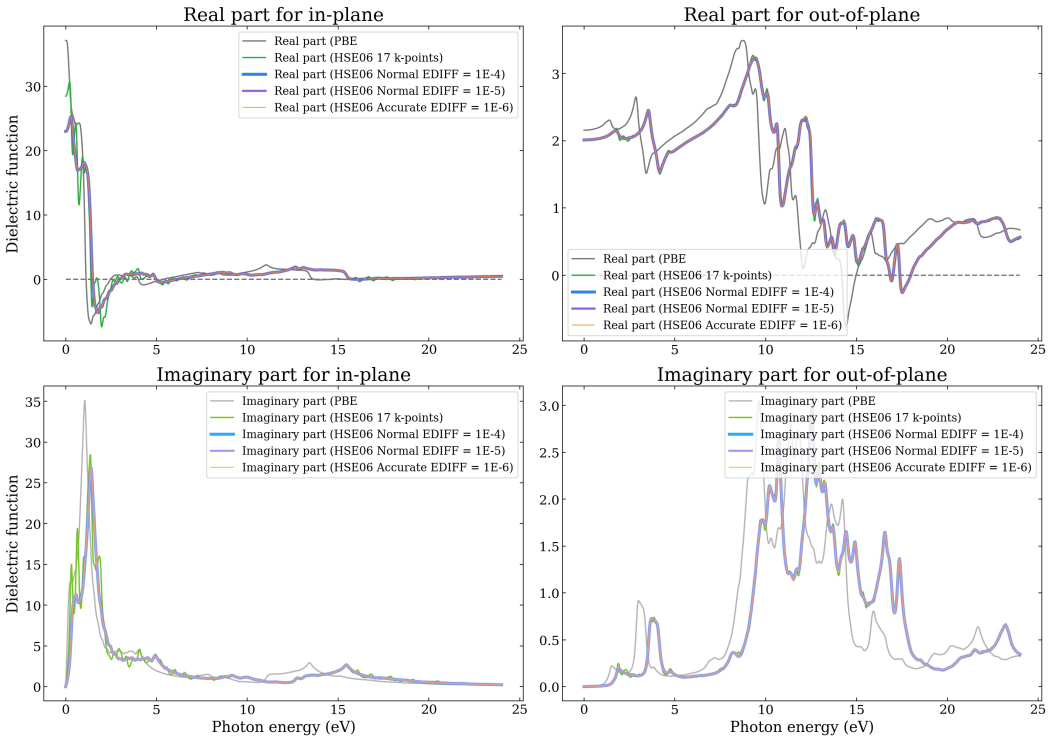

Figure S16: Comparison of PBE and HSE06 functionals for the dielectric function of the Graphene-Borophene bilayer and of the effect of different energy convergence criteria as well as the number of k-points used for the HSE06 calculations, namely $17\times17\times1$ mesh versus a $65\times61\times1$ mesh.

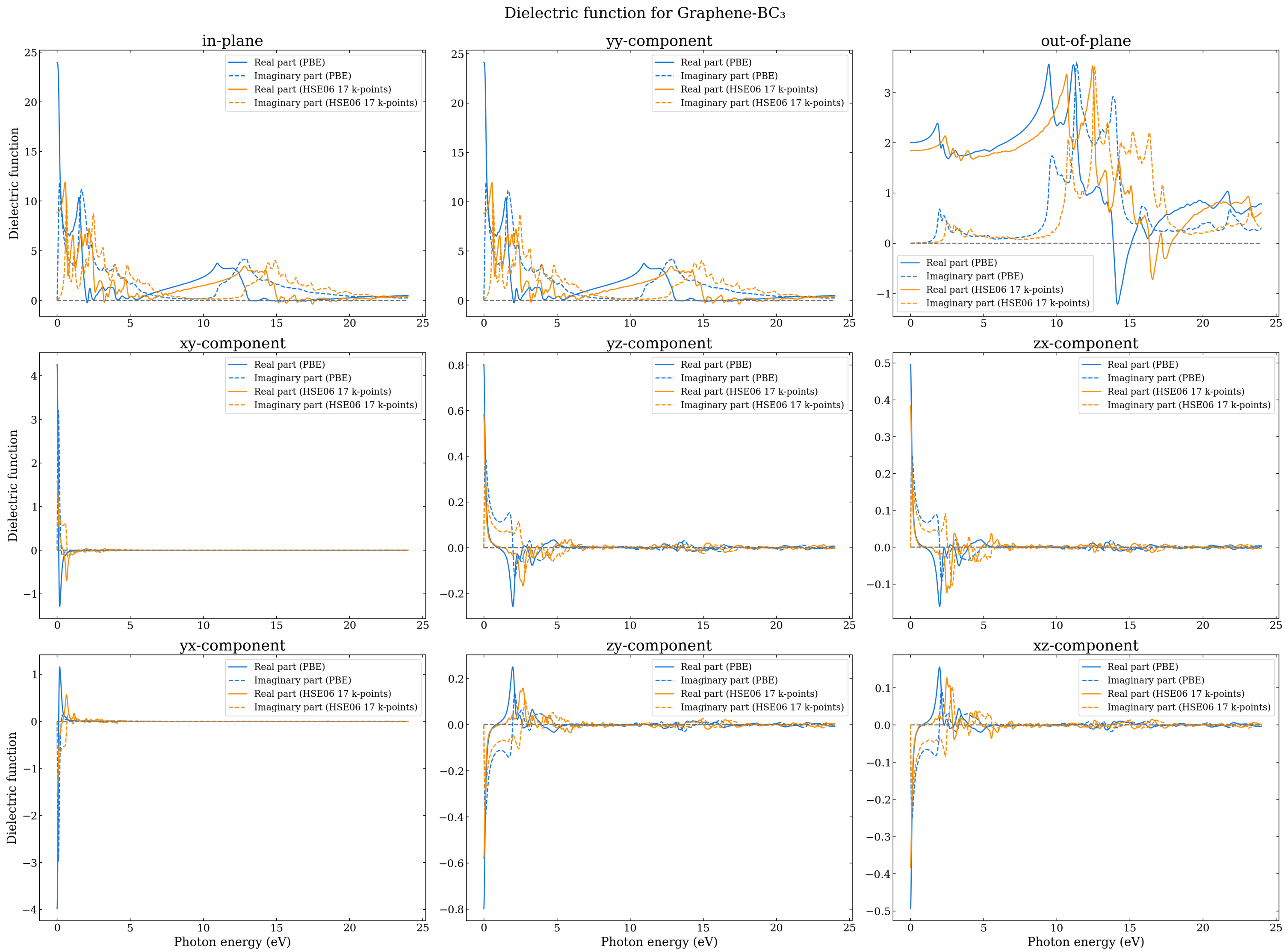

Figure S17: The components of the dielectric function of the Graphene-BC_3 bilayer as calculated using the PBE functional displaying the small, non-zero values of the off-diagonal elements, and the relationships: $yx = -xy$, $yz = -zy$, $yz = -zy$.

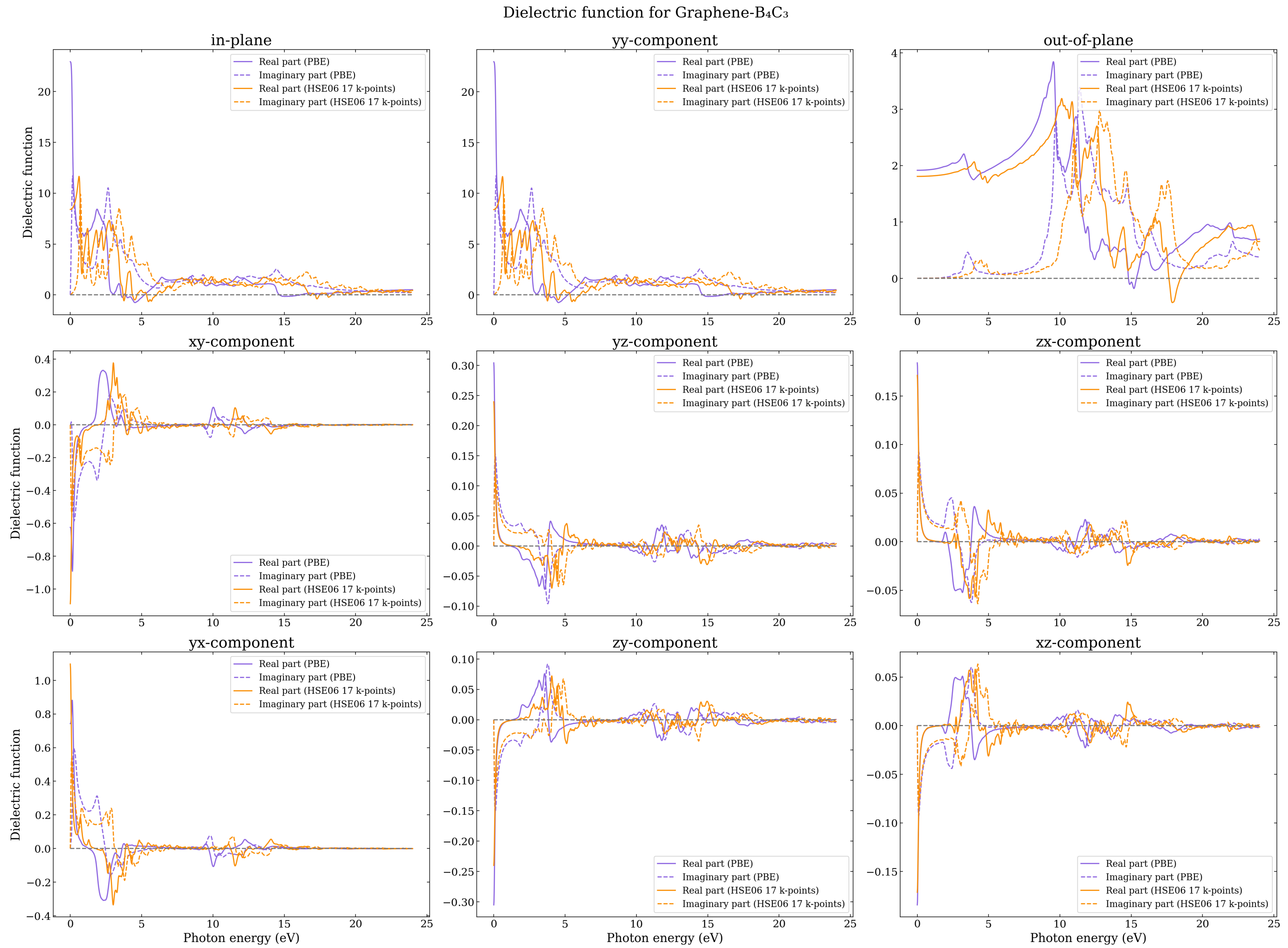

Figure S18: The components of the dielectric function tensor of the Graphene-B_4C_3 bilayer as calculated using the PBE functional displaying the small, non-zero values of the off-diagonal elements, and the relationships: $yx = -xy$, $yz = -zy$, $yz = -zy$.

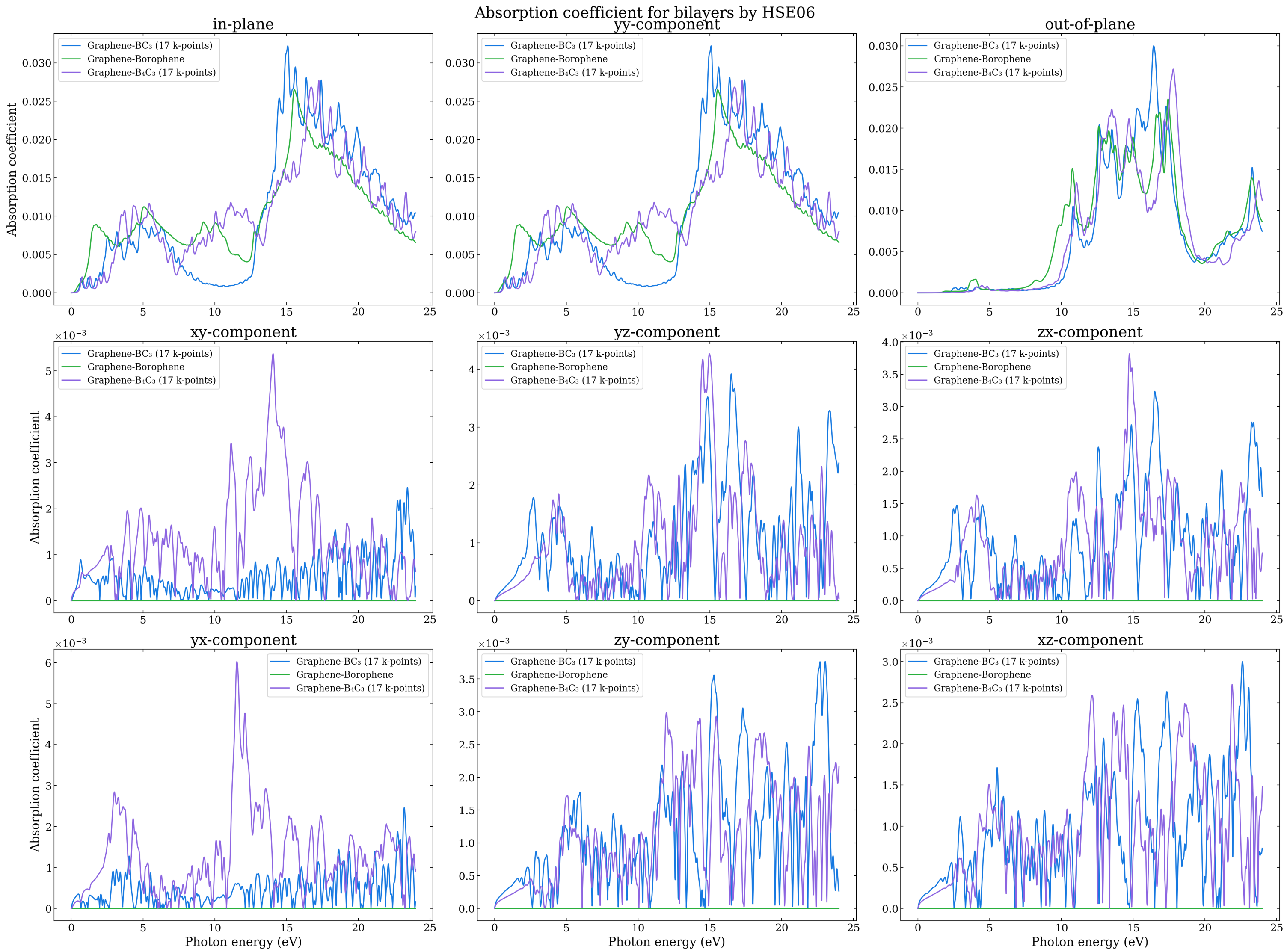

Figure S19: The absorption coefficient for the three bilayer heterostructures as calculated using the HSE06 functional.

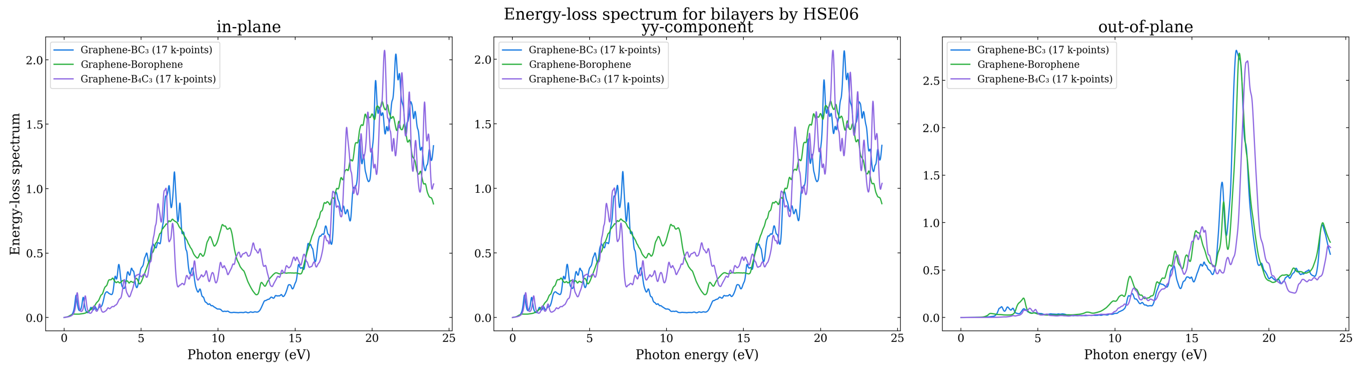

Figure S20: The loss function for the three bilayer heterostructures as calculated using the HSE06 functional.

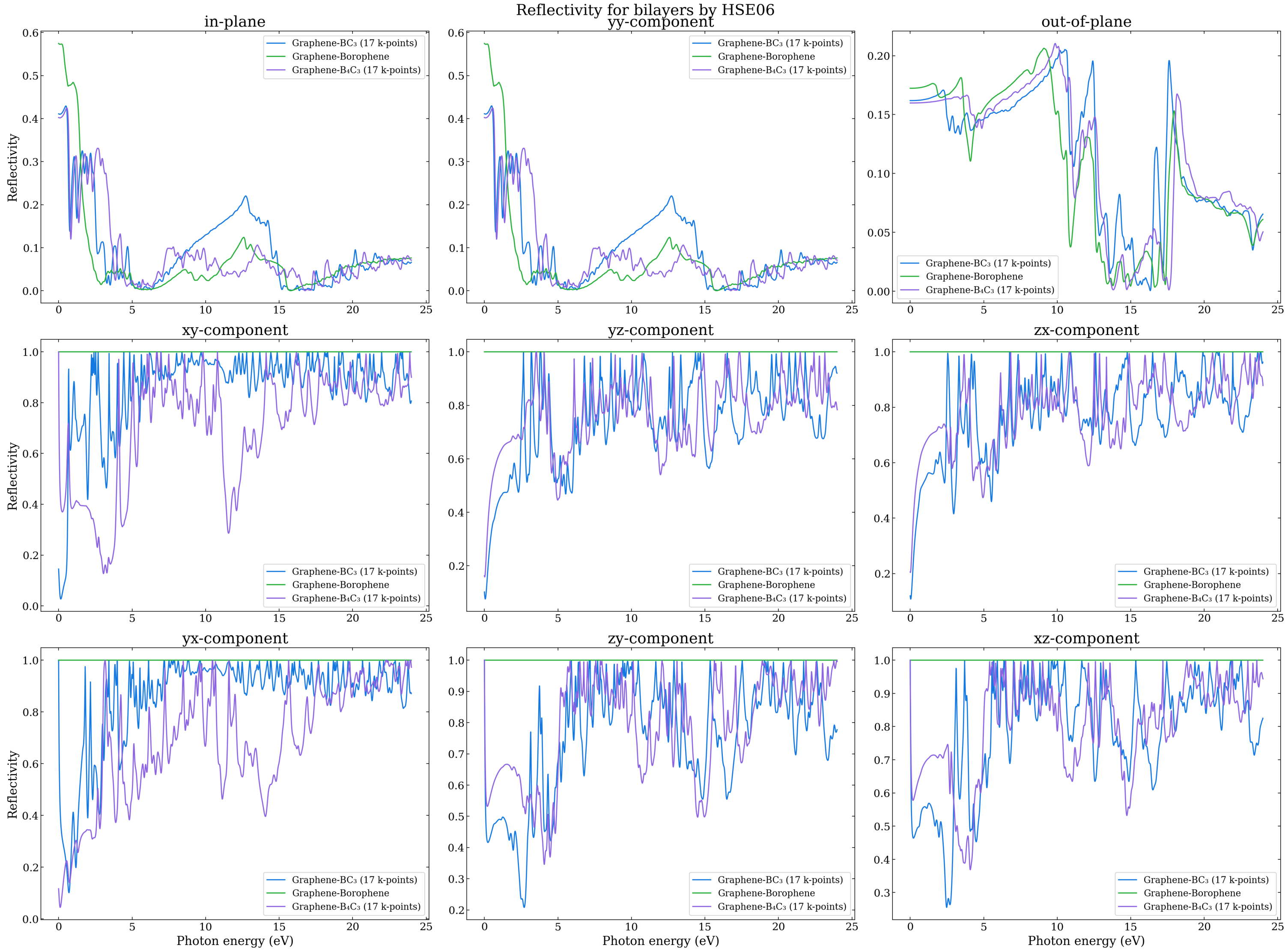

Figure S21: The reflectivity for the three bilayer heterostructures as calculated using the HSE06 functional.

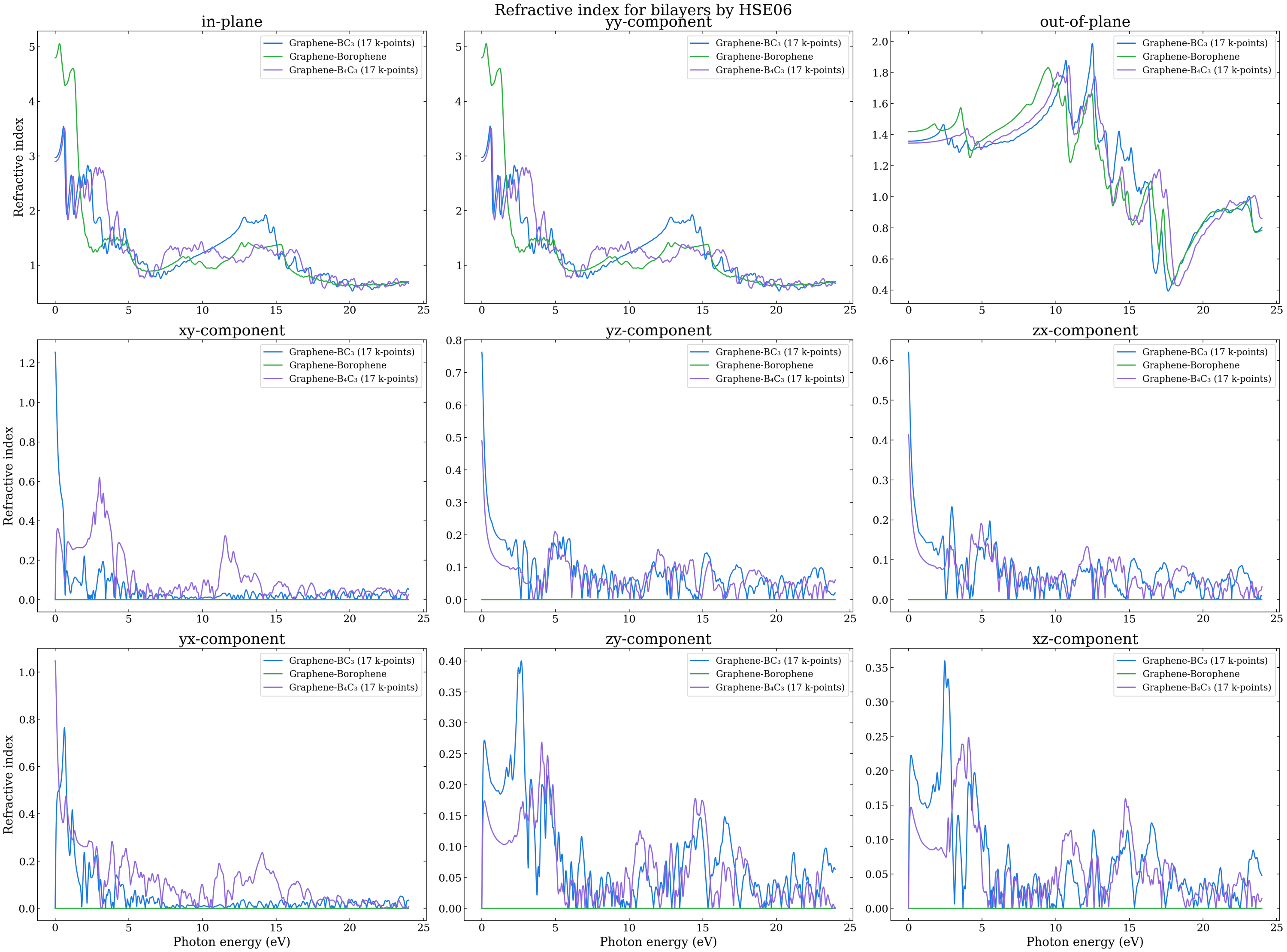

Figure S22: The refractive index for the three bilayer heterostructures as calculated using the HSE06 functional.

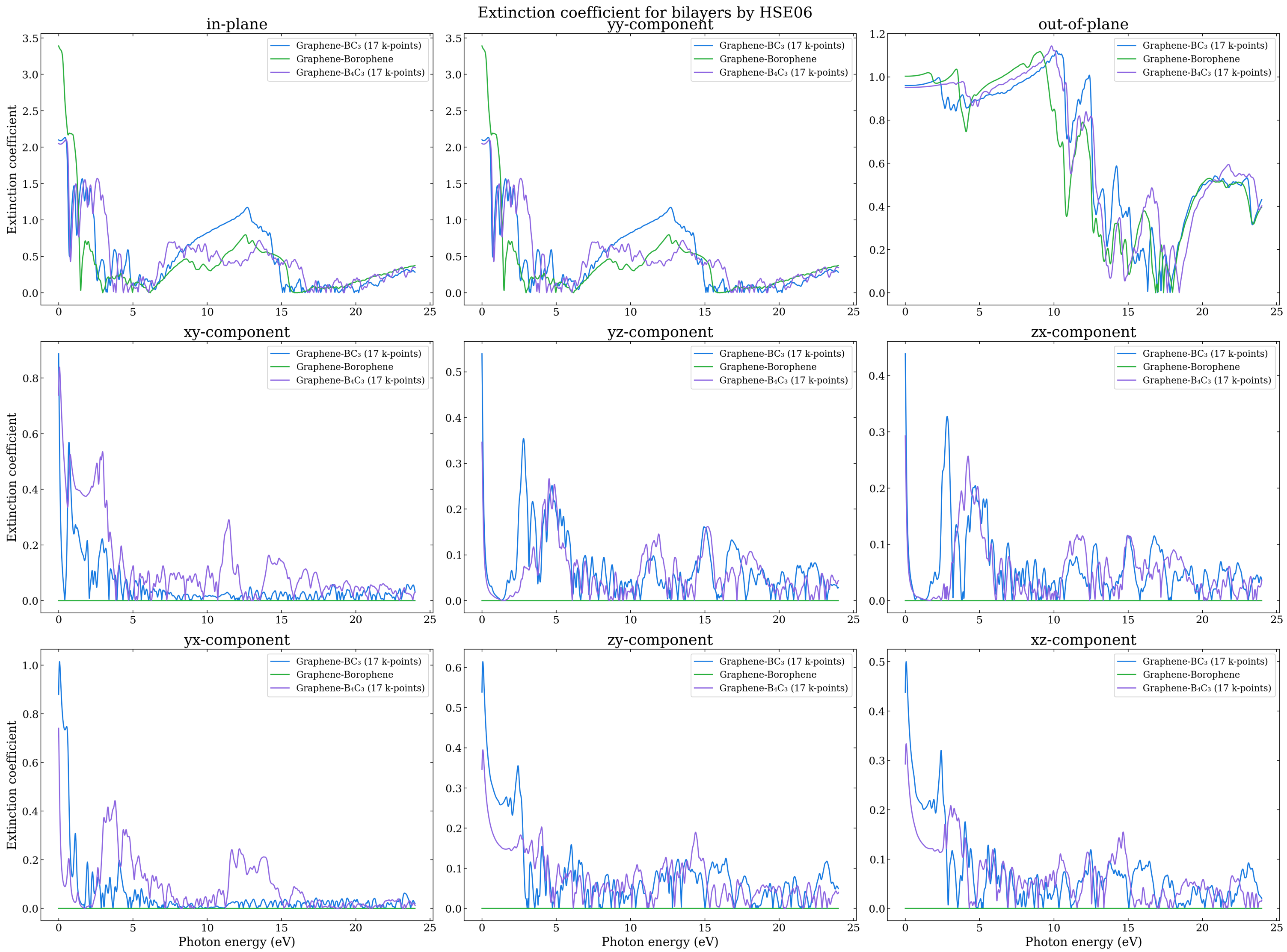

Figure S23: The extinction coefficient for the three bilayer heterostructures as calculated using the HSE06 functional.
